# Tetrandrine augments melanoma cell immunogenicity via dual inhibition of autophagic flux and proteasomal activity enhancing MHC-I presentation

**DOI:** 10.1038/s41401-025-01507-9

**Published:** 2025-02-27

**Authors:** Li-na He, Yu-jiao Liu, Jun-bo Jiang, Ding-ye Wang, Yu-ling Li, Shi-ji Zeng, Zi Guo, Pei-yan Yao, Zi-chang Lin, Si-xian Lv, Xiao-yi Liu, Wei Guo, Fang Liu, Biao-yan Du, Ting-xiu Zhao, Jian-yong Xiao, Ya-fei Shi, Kun Wang

**Affiliations:** 1 Research Center of Integrative Medicine, School of Basic Medical Sciences, Guangzhou University of Chinese Medicine, Guangzhou, 510006 China; 2 Department of Biochemistry, Guangzhou University of Chinese Medicine, Guangzhou, 510006 China; 3 Department of Pathology and Pathophysiology, Guangzhou University of Chinese Medicine, Guangzhou, 510006 China; 4 Department of Human Anatomy, Guangzhou University of Chinese Medicine, Guangzhou, 510006 China; 5 The First Affiliated Hospital of Guangzhou University of Chinese Medicine, Guangzhou University of Chinese Medicine, Guangzhou, 510006 China; 6 School of Pharmaceutical Sciences, Guangzhou University of Chinese Medicine, Guangzhou, 510006 China

**Keywords:** melanoma, tetrandrine, MHC-I, autophagy, proteasome, TPC2 channel

## Abstract

MHC-I-mediated antigen presentation is pivotal in antitumor immunity, enabling the recognition and destruction of tumor cells by CD8^+^ T cells. Both the proteasome and autophagy serve as essential cellular degradation mechanisms that regulate the stability and functionality of MHC-I molecules. In melanoma, modulating the pathways that affect MHC-I antigen presentation is pivotal and can profoundly influence the therapeutic outcomes of immunotherapy. Our initial effort of this study was a screening process to identify natural compounds capable of amplifying MHC-I surface expression on B16 melanoma cells. Utilizing flow cytometry with fluorescently tagged antibodies, we identified tetrandrine (Tet), a bisbenzylisoquinoline alkaloid derived from the root of *Stephania tetrandra*, as a potent enhancer of MHC-I-mediated antigen presentation in B16 melanoma cells. We demonstrate that tetrandrine (2.5, 5, 7.5 μM) dose-dependently upregulates both surface and total MHC-I protein levels in B16 or A375 melanoma cells by simultaneously inhibiting autophagy and proteasomal activity, two key pathways involved in MHC-I degradation. This dual inhibition stabilizes MHC-I molecules, leading to enhanced tumor antigen presentation and improved recognition by CD8^+^ T cells. In co-culture systems, tetrandrine treatment increased CD8^+^ T cell activation and cytotoxicity against melanoma cells, evidenced by elevated IFN-γ secretion and increased tumor cell apoptosis. Administration of tetrandrine (50 mg·kg^-1^·d^-1^, i.g., for 15 days) significantly suppressed melanoma growth in mouse models accompanied by increased CD8^+^ T cell infiltration and activation within the tumor microenvironment. Notably, tetrandrine synergized with anti-PD-1 immune checkpoint therapy, leading to enhanced tumor growth inhibition compared to either treatment alone. We revealed that tetrandrine (7.5 μM) blocked the lysosomal calcium efflux channel TPC2, disrupting lysosomal calcium homeostasis, thus impairing lysosomal acidification and proteasomal activity, thereby stabilizing MHC-I molecules and promoting antigen presentation. These results highlight tetrandrine’s unique mechanism of action in enhancing MHC-I-mediated antigen presentation through dual inhibition of autophagic flux and proteasomal degradation. This study underscores tetrandrine’s potential as a novel immunomodulatory agent to boost CD8^+^ T cell-mediated tumor cell eradication and enhance the efficacy of immune checkpoint therapies.

## Introduction

The intricate interplay between cancer cells and the immune system forms the cornerstone of developing advanced immunotherapies, especially against melanoma—a malignancy renowned for its aggressive behavior and adeptness at evading immune surveillance [1, 2]. The success of these therapies hinges critically on the immune system’s proficiency in recognizing and destroying tumor cells, a process predominantly mediated by Major Histocompatibility Complex class I (MHC-I) molecules [3, 4]. These molecules facilitate the precise presentation of tumor antigens, thereby enabling CD8^+^ T cells—the key players of the adaptive immune response—to accurately identify and target malignant cells [5, 6]. In melanoma, modulating the pathways that affect MHC-I antigen presentation is pivotal and can profoundly influence the therapeutic outcomes of immunotherapy [7, 8].

Both the proteasome and autophagy serve as essential cellular degradation mechanisms that regulate the stability and functionality of MHC-I molecules, critical for the immune system’s capacity to recognize and eliminate malignantly transformed or infected cells through the presentation of endogenous peptides [9, 10]. The proteasome directly modulates the surface levels of MHC-I molecules; its dysregulation or hyperactivity can lead to their augmented degradation, reducing their surface expression and, consequently, diminishing the immune system’s efficacy in recognizing and targeting aberrant cells [11]. On the contrary, proteasome inhibition typically results in the increased presence of MHC-I molecules, thereby boosting their detectability by T cells and enhancing immune surveillance [12].

Similarly, autophagy influences the stability and surface expression of MHC-I molecules [13, 14]. Known primarily for its role in recycling cellular constituents under stress, autophagy can also orchestrate the degradation of MHC-I molecules under specific conditions, thus reducing their surface density and impairing the immune system’s detection capabilities [10, 15]. However, strategic inhibition of autophagy has been noted to elevate MHC-I molecule presence on the cell surface, subsequently enhancing immune recognition and response efficiency [16]. A thorough understanding of the dual roles of the proteasome and autophagy in the regulation of MHC-I degradation provides critical insights necessary for the development of novel therapeutic strategies.

Tetrandrine, a bisbenzylisoquinoline alkaloid derived from the root of *Stephania tetrandra*, is known for its broad pharmacological activities, including anti-inflammatory, antifibrotic, and antitumor effects [17]. In this study, we demonstrate that tetrandrine holds significant potential in enhancing MHC-I-mediated antigen presentation in melanoma cells through its dual modulatory effects on autophagy and proteasome activity. Mechanistically, tetrandrine inhibits autophagic flux by disrupting lysosomal acidification, a process mediated through its blockade of the lysosomal calcium efflux channel TPC2 [18, 19]. This disruption leads to impaired autophagic degradation, stabilizing MHC-I molecules and preventing their lysosomal degradation. Concurrently, tetrandrine suppresses proteasomal activity by reducing cytosolic calcium levels, which are essential for optimal proteasomal function [20]. Together, these dual effects preserve the stability and abundance of MHC-I molecules on the cell surface, significantly enhancing the presentation of antigenic peptides.

This improved antigen presentation boosts the recognition of tumor cells by CD8^+^ T cells, increasing their cytotoxicity and immune-mediated tumor cell eradication. Furthermore, our in vivo studies illustrate that tetrandrine not only inhibits melanoma progression but also promotes CD8^+^ T cell activation and infiltration within the tumor microenvironment. Notably, tetrandrine synergizes with anti-PD-1 therapy, further enhancing tumor suppression and highlighting its potential in combination immunotherapy. By targeting key cellular degradation pathways, tetrandrine offers a novel mechanism-based strategy to enhance MHC-I-mediated antigen presentation and strengthen antitumor immunity, presenting a promising avenue for immunotherapeutic development in melanoma and other cancers.

Overall, our study provides valuable insights into how tetrandrine modulates key cellular degradation pathways to enhance antigen presentation. By elucidating its dual inhibitory effects on autophagy and proteasome activity, we contribute to the broader understanding of strategies to bolster immune system function in the fight against cancer. These findings underscore the potential of targeting cellular processes to improve tumor recognition and immune-mediated destruction. Moreover, tetrandrine’s ability to synergize with existing immunotherapies, such as anti-PD-1 therapy, highlights its therapeutic promise. This study not only offers a foundation for developing more effective immunotherapeutic strategies for melanoma but also opens new avenues for exploring its application across a broader range of cancers.

## Materials and methods

### Reagents and antibodies

Tetrandrine (Cat. No. T2996), the natural product library (Cat. No. L6000), doxycycline hyclate (Cat. No. T1687L), and NAADP antagonist Ned-19 (Cat. No. T12205) were sourced from TargetMol Chemicals (Shanghai, China). Autophagy inhibitors, including bafilomycin A1 (Cat. No. S1413), chloroquine (Cat. No. C6628), proteasome inhibitor MG132 (Cat. No. S2619), and lysosome osmolytic agent Gly-Phe-β-naphthylamide (GPN, Cat. No. S6846) were purchased from Selleckchem (Houston, TX, USA). The TPC2 agonist TPC2-A1-N (Cat. No. HY-131614) was obtained from MedChemExpress (Monmouth, NJ, USA). Primary antibodies against β-actin (Cat. No. 3700), GAPDH (Cat. No. 5174), LC3-I/II (Cat. No. 12741), p62 (Cat. No. 88588), Cathepsin B (Cat. No. 31718), Cathepsin D (Cat. No. 2284), and MHC-I (Cat. No. 35923) were acquired from Cell Signaling Technology (Boston, MA, USA). The primary antibody for HLA-A/B (Cat. No. A8754) was provided by ABclonal (Wuhan, Hubei, China). The antibodies against H2K^b^ (mouse MHC class I) (Cat. No. sc-59199) and mouse Cathepsin D (Cat. No. sc-377299) were sourced from Santa Cruz Biotechnology (Dallas, TX, USA). Secondary antibodies, including HRP-conjugated anti-mouse IgG (Cat. No. AS004), anti-rat IgG (Cat. No. AS0028), and anti-rabbit IgG (Cat. No. AS014), were also obtained from ABclonal (Wuhan, Hubei, China). The pSLIK_Hyg_mTurquoise2_Atg4b^C74A^ plasmid (Addgene, Cat. No. 161733) was obtained from Alec Kimmelman’s lab. The hLAMP1-mCherry (Cat. No. VB900162-5529yba) and the hTPC2-GCaMP6m constructs (Cat. No. VB241204-1512rtc) were provided by VectorBuilder (Guangzhou, China). The GFP-LC3 plasmid was provided by Professor William KK Wu (The Chinese University of Hong Kong). Mouse CD8a (Ly-2) MicroBeads for lymphocyte isolation (Cat. No. 130-117-044) were purchased from Miltenyi Biotec (Bergisch Gladbach, Germany).

### Cell culture

B16-F10 (B16) melanoma cells were procured from the American Type Culture Collection (ATCC, Rockville, MD, USA), while A375 human melanoma cells were sourced from Wuhan Pricella Biotechnology. B16 cells stably expressing ovalbumin (OVA) were acquired from Beijing Crispr Biotechnology Co., Ltd. Both B16-F10 and A375 melanoma cell lines were cultured in RPMI-1640 medium or Dulbecco’s Modified Eagle's Medium (DMEM), supplemented with 10% fetal bovine serum (FBS), 100 U/mL penicillin, and 100 μg/mL streptomycin. The culture medium was refreshed every 2-3 days, and cells were passaged at 70%–80% confluence using 0.25% trypsin-EDTA. Cells were maintained in a humidified atmosphere at 37°C with 5% CO_2_ to ensure optimal growth conditions. For experimental treatments, cells were seeded at an appropriate density in culture plates and allowed to adhere for 24 h before any reagents were added.

### Flow cytometry analysis

Following treatment, B16 and A375 cells were harvested and washed twice with cold phosphate-buffered saline (PBS). The cells were then incubated with Pacific Blue™ anti-mouse H2K^b^ (BioLegend, Cat. No. 116514), FITC anti-mouse H2K^b^ (BD Biosciences, Cat. No. 553569), and OVA257-264 peptide bound to H2K^b^ (BioLegend, Cat. No. 141605) for 30 min at 4°C in the dark to prevent photobleaching and ensure optimal staining. After staining, the cells were washed twice with PBS to remove any unbound antibodies and resuspended in flow cytometry buffer (PBS containing 2% FBS). Flow cytometric analysis was performed using a BD LSR Fortessa flow cytometer. The data were analyzed using FlowJo software (Tree Star, Ashland, OR, USA).

### Western blot analysis

After treatment, cells were lysed in RIPA buffer containing protease and phosphatase inhibitors. Protein concentration was determined using the BCA Protein Assay. Equal amounts of protein were separated on SDS-PAGE gels and transferred to PVDF membranes. Membranes were blocked with 5% non-fat milk in TBST for 1 h at room temperature, then incubated overnight at 4°C with primary antibodies. After washing, membranes were incubated with HRP-conjugated secondary antibodies for 1 h at room temperature. Bands were visualized using the Tanon™ 5200CE Chemi-Image System and quantified using GelPro software.

### mCherry-GFP-LC3 autophagy flux assay

A375 cells were transfected with the pBABE-puro mCherry-GFP-LC3B plasmid (Addgene, Cat. No. 22418) and subsequently treated with indicated compounds to assess autophagic flux. After treatment, the cells were washed with PBS and fixed with 4% PFA (paraformaldehyde) for 15 min at room temperature. Once fixed, the cells were washed again, stained with DAPI, and analyzed for autophagic flux using an LSM 800 confocal microscope (Carl Zeiss, Jena, Germany). This confocal microscopy technique allowed for the imaging of cells, capturing both mCherry and GFP fluorescence. Normally, autophagosomes are visualized as yellow puncta (GFP^+^mCherry^+^) due to the co-localization of GFP and mCherry, whereas autolysosomes, formed by the fusion of autophagosomes with lysosomes, appear as red puncta (mCherry^+^) because the GFP signal is quenched in the acidic lysosomal environment. The inhibition of autophagic flux leads to an accumulation of yellow puncta (GFP^+^mCherry^+^).

### Electron microscopy for autophagosome-lysosome fusion

To assess autophagosome-lysosome fusion, A375 cells were treated with tetrandrine and prepared for transmission electron microscopy (TEM). After treatment, cells were washed with PBS and collected. Subsequently, the cell pellets were fixed with 2.5% glutaraldehyde in the dark at room temperature for 30 min. Following this, cells were post-fixed in 1% osmium tetroxide, dehydrated through a graded ethanol series, and embedded in epoxy resin. Ultrathin sections (70–90 nm) were cut using an ultramicrotome and mounted on copper grids. The samples were then examined using a transmission electron microscope to observe autophagosomes and autolysosomes. Autophagosomes are characterized as double-membrane vesicles containing partially degraded cellular components, while autolysosomes are identified as single-membrane vesicles with denser contents due to lysosomal degradation.

### Crystal violet cytotoxicity assay

Following co-culture of tetrandrine-pretreated B16-OVA cells with CD8^+^ T cells, cytotoxicity was assessed using a crystal violet staining method. Post co-culture, cells were washed twice with PBS to remove non-adherent cells and debris. The remaining adherent cells were fixed with 4% PFA for 15 min at room temperature. After fixation, cells were stained with 0.5% crystal violet solution for 20 min. Crystal violet binds to cellular DNA and proteins, providing a measure of cell density. Excess stain was carefully washed off with distilled water until the rinse was clear, ensuring only adherent cells were stained. The plates were then air-dried before quantifying cell viability, after which the bound dye was solubilized by adding 1% acetic acid and gently shaking the plates for 10 min. The absorbance of the resulting solution was measured at 595 nm using a microplate reader. Absorbance values were directly proportional to the number of viable cells, providing an indication of the cytotoxic effects induced by CD8^+^ T cells on tetrandrine-pretreated B16-OVA cells.

### ELISA for IFN-γ release

Supernatants from co-cultured tetrandrine-pretreated B16-OVA cells and CD8^+^ T cells were collected to measure IFN-γ levels using a specific ELISA kit (Cat. No. RK00019, ABclonal). The assay was performed following the manufacturer’s instructions. Briefly, 96-well plates pre-coated with anti-IFN-γ antibodies were used. Collected supernatants were added to the wells and incubated at room temperature. After washing, a biotinylated detection antibody was added, followed by streptavidin-HRP conjugate. A substrate solution was then added for colorimetric detection of IFN-γ. The reaction was stopped with a stop solution, and the optical density was read using a microplate reader. Controls included supernatants from untreated co-cultures and media alone.

### Assessing CD8^+^ T cell-mediated cytotoxicity using flow cytometry

OT-I mice, which possess T-cell receptors specific for OVA^257–264^ presented by H2K^b^, were sourced from GENEANDPEASE (Yangzhou, China). Spleens were harvested, mechanically dissociated, and lymphocytes were isolated using a lymphocyte separation solution (Cat. No. 7211011, Dakewe Biotech Co., Ltd.). CD8^+^ T cells were enriched by incubating with Mouse CD8a (Ly-2) MicroBeads (Cat. No. 130-117-044, Miltenyi) and MACS buffer (Cat. No. 130-091-222-1, Miltenyi) at 4 °C for 10 min, followed by magnetic separation. Cells were then resuspended in complete medium supplemented with IL-2 (Cat. No. 212-12-100, Peprotech, Cranbury, NJ, USA) for subsequent experiments. To evaluate CD8^+^ T cell-mediated cytotoxicity, B16-OVA melanoma cells pretreated with tetrandrine were co-cultured with CD8^+^ T cells derived from OT-I mice. Post co-culture, cells were harvested and stained with APC-conjugated anti-mouse CD8 antibody (Cat. No. 100712, Biolegend, San Diego, CA, USA) and FVS620 viability dye (Cat. No. 553142, BD Biosciences, San Jose, CA, USA) to differentiate between live and dead cells. Flow cytometry was used to analyze the samples. Dead melanoma cells were identified as CD8^-^ FVS620^+^ populations. This gating strategy specifically quantified the population of dead melanoma cells, thus assessing the cytotoxic impact of CD8^+^ T cells on tetrandrine-pretreated melanoma cells. The percentage of dead melanoma cells in each condition was calculated to gauge the effectiveness of tetrandrine in enhancing T cell-mediated killing of melanoma cells.

### Assessment of lysosomal acidification using lysotracker

To assess lysosomal acidification, A375 cells were seeded onto glass slides in 12-well plates and treated with various compounds. The cells were then incubated at 37°C in the dark with LysoTracker Red (50 nM, Cat. No. L7528, Thermo Fisher Scientific), a fluorescent dye that selectively accumulates in acidic compartments. After 20 min, the cells were washed with PBS and visualized using a laser confocal scanning microscope. Images were captured to analyze fluorescent intensity using ImageJ software. Untreated cells served as control samples to establish baseline levels of lysosomal acidification.

### Proteasome 20S activity assay

The proteasome 20S activity assay was performed using the Amplite® Fluorimetric Proteasome 20S Activity Assay Kit (AAT Bioquest, Cat. No. 13456). This assay quantifies the chymotrypsin-like protease activity of the proteasome with the fluorogenic substrate LLVY-R110, which releases the green fluorescent compound R110 upon cleavage, monitored at Ex/Em = 490/525 nm. B16 cells were seeded at a density of 8000 cells per well in a 96-well plate with black walls and a clear bottom. After allowing the cells to adhere, the cells were treated with indicated compounds for 6 h. Following treatment, the proteasome working solution was added to each well. The plate was then incubated at 37°C in a 5% CO_2_ atmosphere for at least 2 h, protected from light. According to the manufacturer’s instructions, fluorescence intensity was measured using a fluorescence microplate reader.

### Intracellular Ca^2+^ imaging

A375 cells were seeded in confocal dishes, allowed to adhere overnight, and treated with control, Tetrandrine (Tet), TPC2-A1-N (TPC2 agonist), or Ned-19 (NAADP antagonist). After treatment, cells were washed with PBS, stained with 3 μM Fluo-4/AM (Invitrogen, Cat. No. F14201) at 37°C for 30 min, and imaged using an an LSM 800 confocal microscope (Carl Zeiss, Jena, Germany). Cytoplasmic Ca²^+^ levels were assessed by comparing Fluo-4 fluorescence intensity at 506 nm emission among groups. For lysosomal Ca^2+^ monitoring, cells were pretreated with control, BAPTA (Ca²^+^ chelator, BAPTA-AM, Thermo Fisher Scientific, Cat. No. B1205), or Tet, followed by PBS washing and Fluo-4/AM staining. Time-lapse confocal imaging was performed (one frame every 5 s, total duration 400 s). At the 15th frame, the lysosome osmolytic agent GPN (400 µM) was added to increase lysosomal membrane permeability, inducing lysosomal Ca^2+^ release. Fluorescence changes were quantified as Δ*F*/*F*_0_, where *F*_0_ represents the baseline fluorescence before stimulation and Δ*F* is the change in fluorescence intensity over time, normalized to *F*_0_. For lysosomal TPC2 Ca^2+^ release assays, a stable A375 cell line expressing hTPC2-GCaMP6m was used. Cells were pretreated with control, BAPTA, or Tet. Lysosomal TPC2 Ca²^+^ release was triggered by the addition of TPC2-A1-N (30 μM) at the 8th frame during time-lapse confocal imaging (5 s/frame, total duration 600 s). Changes in lysosomal Ca²^+^ levels were reflected by alterations in TPC2-GCaMP6m fluorescence intensity. Fluorescence changes were quantified as Δ*F*/*F*_0_. ImageJ software was used for fluorescence intensity measurements and data analysis.

### In vivo melanoma model

Eight-week-old C57BL/6 mice were obtained from the Guangdong Provincial Medical Laboratory Animal Center and acclimated in a SPF facility. The mice were subcutaneously injected with 2 × 10^5^ B16 melanoma cells to establish tumors. Before cell injection, the mice were randomly divided into different treatment groups (*n* = 5 per group). Mice received daily oral gavage treatments of tetrandrine at concentrations of 0, 25, 50, and 75 mg/kg. Tumor growth was monitored every day using calipers, and tumor volume was calculated using the formula: Volume = Length × Width^2^/2. At the end of the experiment, tumors were harvested and weighed. For additional experiments, mice were grouped as follows: control, αCD8 (200 μg/mouse, i.p., every 3 days), tetrandrine (50 mg/kg), and αCD8 + tetrandrine; or control, αPD-1 (200 μg/mouse, i.p., as per schedule), tetrandrine (50 mg/kg), and αPD-1 + tetrandrine. The antibodies for in vivo PD-L1 blockade (Cat. No. A2122) and CD8^+^ T cell depletion (Cat. No. A2102) were purchased from Selleckchem (Houston, TX, USA). This study demonstrated that tetrandrine significantly inhibits melanoma growth and enhances the efficacy of anti-PD-1 therapy through CD8^+^ T cell-mediated immune responses. All procedures involving animals were conducted in strict accordance with ethical standards and were formally approved by the Animal Ethics Committee of Guangzhou University of Chinese Medicine (Approval No. 20240427005).

### Immunohistochemistry and immunofluorescence staining

Excised tumors were fixed in 4% PFA, embedded in paraffin, and sectioned at a thickness of 5 µm. For immunohistochemistry, sections were deparaffinized, rehydrated, and subjected to antigen retrieval using a citrate buffer (pH 6.0) in a microwave. Endogenous peroxidase activity was quenched with 3% hydrogen peroxide. The sections were then blocked with 5% bovine serum albumin (BSA) for one hour at room temperature. A primary antibody against CD8 (Thermo Fisher Scientific, Cat. No. 14-0081-85, 1:200 dilution) was applied and left overnight at 4°C. After PBS washes, the sections were incubated with an HRP-conjugated secondary antibody for one hour at room temperature. The signal was developed using a DAB substrate kit (ZSGB-Bio, Cat. No. ZLI-9018), and the sections were counterstained with hematoxylin. Images were captured using a light microscope.

For immunofluorescence staining, the sections underwent similar processing steps for deparaffinization, rehydration, and antigen retrieval. After blocking with 5% BSA, the sections were incubated overnight at 4°C with a primary antibody against CD8 (1:200 dilution). Following PBS washes, the sections were incubated with an Alexa Fluor® 594-conjugated anti-rat secondary antibody (Abcam, Cat. No. ab150160) for one hour at room temperature in the dark. Nuclei were counterstained with DAPI. Fluorescence images were acquired using an LSM 800 confocal microscope (Carl Zeiss, Jena, Germany).

### Statistical analysis

All statistical analyses were performed using GraphPad Prism software (GraphPad Software, San Diego, CA, USA). Data are presented as mean ± standard deviation (SD) or standard error of the mean (SEM) from at least three independent experiments. Comparisons between two groups were conducted using the Student’s *t*-test, while one-way ANOVA followed by Tukey’s multiple comparison test was employed for comparisons among multiple groups. A *P*-value of less than 0.05 was considered statistically significant. The specific statistical test used for each analysis is indicated in the figure legends, ensuring clarity and transparency in data interpretation.

## Results

### Tetrandrine enhances MHC-I-mediated antigen presentation in melanoma cells

**Fig. 1:**
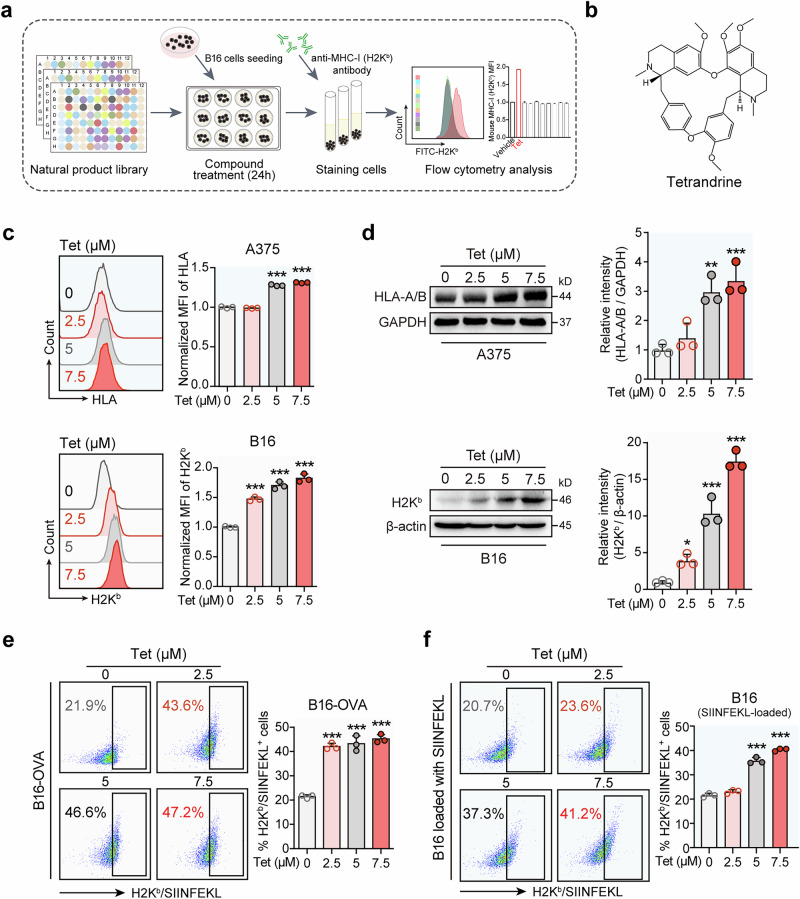
Tetrandrine enhances MHC-I-mediated antigen presentation in melanoma cells. **a** Schematic representation of the screening for natural product small molecules that increase MHC-I on the surface of melanoma cells. Using flow cytometry with fluorescently labeled antibodies, we screened for natural products from the TargetMol natural product library that enhance the expression of H2K^b^ (MHC-I) on the surface of B16 melanoma cells. **b** Chemical structure of tetrandrine. **c** Concentration-dependent increase of MHC-I on the surface of melanoma cells by tetrandrine. B16 or A375 melanoma cells were treated with a gradient of tetrandrine concentrations (0, 2.5, 5, 7.5 μM) for 24 h, followed by flow cytometric analysis using an H2K^b^ (HLA-A/B) fluorescent antibody to assess the effect of tetrandrine on the surface expression of MHC-I. **d** Concentration-dependent increase of MHC-I on the surface of melanoma cells by tetrandrine. B16 or A375 melanoma cells were treated with a gradient of tetrandrine concentrations (0, 2.5, 5, 7.5 μM) for 24 h, followed by Western blot analysis using an H2K^b^ (HLA-A/B) antibody to assess the effect of tetrandrine on the total expression of MHC-I. **e** Concentration-dependent enhancement of MHC-I-mediated antigen presentation in melanoma cells by tetrandrine. B16-OVA cells (B16 melanoma cells stably expressing OVA protein) were treated with a gradient of tetrandrine concentrations (0, 2.5, 5, 7.5 μM) for 24 h. Subsequently, flow cytometry was used to quantify the cell surface H2K^b^/SIINFEKL complexes. **f** Concentration-dependent enhancement of MHC-I-mediated antigen presentation in melanoma cells by tetrandrine. B16 cells were incubated with the SIINFEKL peptide and treated with a range of tetrandrine concentrations (0, 2.5, 5, 7.5 μM) for 24 h. Following treatment, flow cytometry was performed to quantify the cell surface H2K^b^/SIINFEKL complexes. Tet, tetrandrine. **P < 0.05, ***P < 0.001 indicate levels of statistical significance.

Our initial efforts involved a screening process to identify natural compounds capable of amplifying MHC-I surface expression on B16 melanoma cells. By utilizing flow cytometry with fluorescently tagged antibodies, we identified tetrandrine from the TargetMol natural product library as a potent enhancer of H2K^b^ (MHC-I) expression, as delineated in Fig. 1a. The molecular structure of tetrandrine is shown for reference (Fig. 1b). Subsequent analyses demonstrated a concentration-responsive enhancement of MHC-I on the surfaces of both B16 and A375 melanoma cell lines following tetrandrine treatment. Employing flow cytometric analysis after administering varying tetrandrine concentrations (0, 2.5, 5, 7.5 μM) for 24 h elucidated a notable amplification in MHC-I expression, escalating with increased tetrandrine dosage (Fig. 1c). To correlate the surface upsurge in MHC-I with its total cellular levels, we conducted Western blot analyses. These assays corroborated that tetrandrine administration proportionately elevated total MHC-I protein levels within both cell lines, mirroring the trends observed in surface expression (Fig. 1d). The functional significance of MHC-I upregulation was investigated using B16-OVA cells, which express the OVA protein, and B16 cells incubated with the SIINFEKL peptide, serving as antigen presentation models. Post tetrandrine treatment, flow cytometry was employed to detect H2K^b^/SIINFEKL complexes on the cell surface. The results indicated a concentration-dependent bolstering in MHC-I-mediated antigen presentation, signifying that tetrandrine not only uplifts MHC-I expression but also substantially enhances antigen presentation to the immune machinery (Fig. 1e, f). Collectively, these insights advocate tetrandrine’s potential as a promising agent to elevate melanoma cell immunogenicity, potentially optimizing the efficacy of immune-centric therapeutic interventions against melanoma.

### Tetrandrine treatment enhances CD8^+^ T cell recognition and killing of melanoma cells

**Fig. 2:**
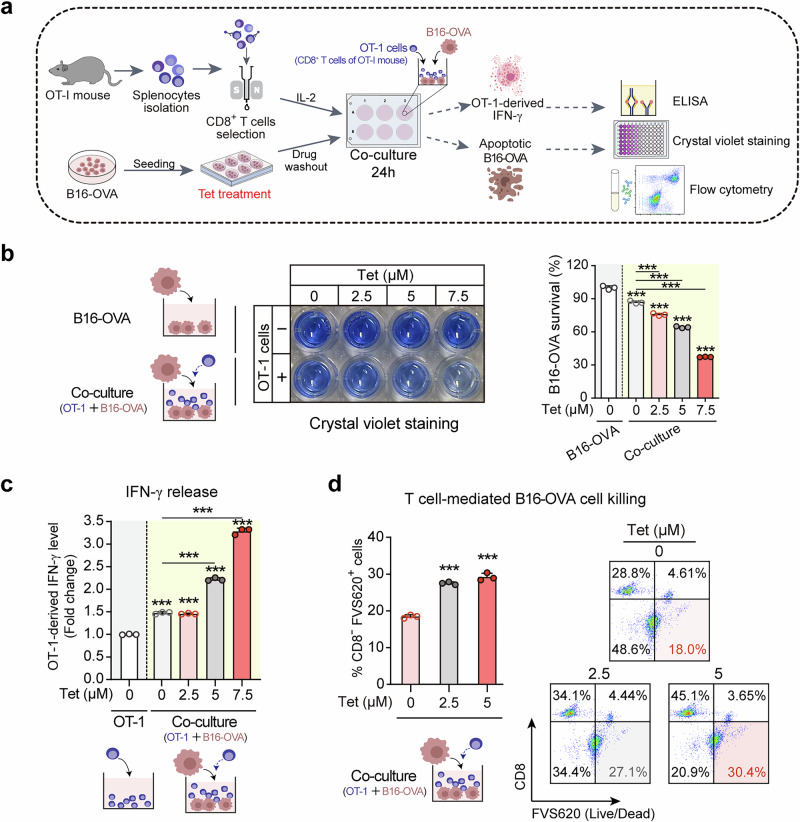
Tetrandrine treatment enhances CD8^+^ T cell recognition and killing of melanoma cells. **a** Schematic of the in vitro immune cytotoxicity assay. B16-OVA cells pretreated with a gradient of tetrandrine concentrations (0, 2.5, 5, 7.5 μM) for 24 h were co-cultured with CD8^+^ T cells from OT-I mice at a 1:10 ratio. After 24 h, CD8^+^ T cell activation and their cytotoxic effect on melanoma cells were assessed by crystal violet staining, ELISA for IFN-γ release, and flow cytometry analysis of B16-OVA cell apoptosis. **b** Tetrandrine enhances the cytotoxicity of CD8^+^ T cells against B16-OVA melanoma cells (crystal violet staining). Results indicate that tetrandrine alone does not have a significant cytotoxic effect on melanoma cells. However, in the presence of CD8^+^ T cells, tetrandrine enhances the cytotoxic effect in a concentration-dependent manner. **c** Tetrandrine treatment increases IFN-γ release by CD8^+^ T cells. Following treatment as depicted, IFN-γ release was measured by ELISA. **d** Tetrandrine enhances the killing of melanoma cells by CD8^+^ T cells (flow cytometry - proportion of APC-CD8(-) FVS620(+) cells). After treatment as shown, the death of B16-OVA cells was assessed by flow cytometry, following co-staining with CD8 antibody and FVS620. Cells negative for CD8 and positive for FVS620 were identified as dead B16-OVA cells. Tet tetrandrine. ***P < 0.001 indicate levels of statistical significance.

Expanding upon our initial discovery that tetrandrine amplifies MHC-I-mediated antigen presentation in melanoma cells, we delved into its consequential effects on CD8^+^ T cell recognition and cytotoxic activity against these cancer cells (Fig. 2). Firstly, we assessed the cytotoxic effects of tetrandrine on melanoma cells using the CCK8 assay, which revealed that tetrandrine itself did not induce cytotoxicity in melanoma cells (Fig. S1). Following this, we conducted an in vitro immune cytotoxicity assay where B16-OVA cells, pretreated with increasing doses of tetrandrine, were co-cultured with CD8^+^ T cells derived from OT-I mice (Fig. 2a). This experimental design aimed to elucidate the influence of tetrandrine-enhanced MHC-I expression on the T cell-mediated destruction of melanoma cells. Our observations revealed a substantial enhancement in T cell-mediated cytotoxicity against tetrandrine-treated B16-OVA cells. This effect was quantitatively assessed via crystal violet staining, demonstrating a direct correlation between tetrandrine concentration and the degree of T cell cytotoxicity, thereby suggesting that tetrandrine’s upregulation of MHC-I expression strengthens T cell recognition and subsequent elimination of melanoma cells (Fig. 2b). Beyond cytotoxicity, tetrandrine’s modulation of CD8^+^ T cell functionality was indirectly assessed by measuring IFN-γ release. When CD8^+^ T cells were co-cultured with tetrandrine-preconditioned melanoma cells, we noted a significant rise in IFN-γ secretion, as determined by ELISA, suggesting that tetrandrine’s enhancement of antigen presentation on melanoma cells indirectly enhances CD8^+^ T cell activation and their effector functions, resulting in an intensified immune response (Fig. 2c). To precisely quantify T cell-mediated killing of melanoma cells, we employed flow cytometry, identifying a marked increase in the proportion of apoptotic melanoma cells (APC-CD8(-) FVS620(+)) in the presence of tetrandrine-treated cells (Fig. 2d). This observation reinforces the premise that tetrandrine elevates the immunogenicity of melanoma cells, thereby facilitating more potent CD8^+^ T cell-mediated cytotoxicity. Collectively, these insights highlight tetrandrine’s promising role in augmenting the effectiveness of T cell-based immunotherapies, enhancing melanoma cell visibility to the immune system, and amplifying CD8^+^ T cell cytotoxic functions.

### Tetrandrine inhibits the late stage of autophagic flux in melanoma cells

**Fig. 3:**
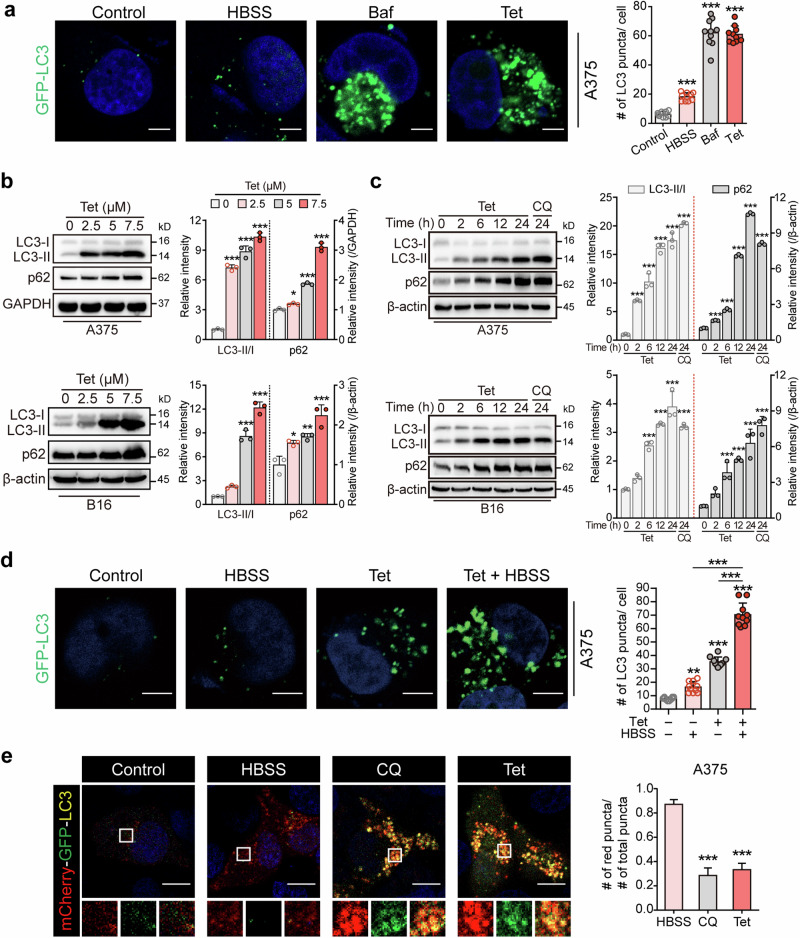
Tetrandrine inhibits the late stage of autophagic flux in melanoma cells. **a** Treatment with tetrandrine increases the number of autophagosomes in melanoma cells. A375-GFP-LC3 cells were treated with vehicle, tetrandrine (5 μM) for 24 h, HBSS (to induce autophagy) for 6 h, or Baf (20 nM) for 24 h, followed by confocal microscopy to count green fluorescent puncta. Scale bar, 10 μm. **b** Tetrandrine induces a concentration-dependent increase in P62 and LC3-II in melanoma cells. A375 and B16 cells were exposed to varying concentrations of tetrandrine (0, 2.5, 5, 7.5 μM) for 24 h. Subsequently, proteins were harvested for Western blot analysis. **c** Time-dependent elevation of P62 and LC3-II is observed with tetrandrine treatment. A375 or B16 melanoma cells were treated with tetrandrine (5 μM) for intervals up to 24 h, followed by protein isolation and Western blot assessment, with CQ (chloroquine, 20 μM) serving as a positive control. **d** Combined HBSS and tetrandrine treatment exacerbates autophagosome accumulation. A375-GFP-LC3 cells underwent treatments with vehicle, HBSS for 6 h, tetrandrine (5 μM) for 24 h, or tetrandrine for 18 h followed by HBSS for the remaining 6 h with continuous presence of tetrandrine. Subsequent analysis of green fluorescent puncta was performed via confocal microscopy. Scale bar, 10 μm. **e** Tetrandrine treatment enhances the colocalization of red and green fluorescence in mCherry-GFP-LC3-transfected melanoma cells, indicating a blockade at the late stage of autophagic flux. After transfection with mCherry-GFP-LC3 plasmids, A375 cells were treated with vehicle, HBSS for 6 h, tetrandrine (5 μM) for 24 h, or CQ (20 μM) for 24 h, followed by confocal microscopy to assess the presence of yellow puncta, signifying the impaired late stage of autophagic flux. Scale bar, 10 μm. Tet tetrandrine, CQ chloroquine, Baf bafilomycin A1, HBSS Hanks’ Balanced Salt Solution. **P < 0.01, ***P < 0.001 indicate levels of statistical significance.

Our results indicate that tetrandrine does not affect the expression levels of genes responsible for MHC-I (human: HLA-A and HLA-B; mouse: H2-K1 and H2-D1) (Fig. S2), indirectly suggesting that tetrandrine elevates MHC-I levels by inhibiting its degradation. Autophagy has been implicated as a critical pathway for the degradation of MHC-I, facilitating immune escape in various cancers, including pancreatic cancer where it promotes MHC-I degradation [16, 21, 22]. This context sets the stage for examining whether tetrandrine enhances MHC-I expression in melanoma cells by modulating autophagy. Employing A375-GFP-LC3 melanoma cells enabled us to visually track autophagosome accumulation and analyze the dynamics of autophagic flux following tetrandrine administration. Initial observations highlighted a pronounced increase in autophagosome numbers in A375-GFP-LC3 cells treated with tetrandrine, as demonstrated by an accumulation of green fluorescent puncta, visible under confocal microscopy (Fig. 3a). This augmentation was compared to cells treated with HBSS, an autophagy inducer, and chloroquine (CQ), a well-known inhibitor of late-stage autophagic flux, which served as comparative controls. Subsequent Western blot analyses elucidated a concentration-dependent rise in P62 and LC3-II levels within A375 and B16 cells treated with tetrandrine, revealing an accumulation of these autophagosome markers (Fig. 3b). These findings suggest that tetrandrine interferes with the progression or degradation of autophagosomes, resulting in their accumulation. A time-resolved study reinforced these observations, revealing a consistent increase in P62 and LC3-II levels within 24 h post-tetrandrine treatment, underscoring the time-dependent impact of tetrandrine on autophagic flux (Fig. 3c).

Exploring further, we assessed the combined effect of HBSS-induced autophagy and tetrandrine treatment, noting a significant amplification in autophagosome aggregation. This synergy implies that tetrandrine predominantly obstructs the late stage of autophagy (Fig. 3d). To further clarify this, we used A375 cells transfected with mCherry-GFP-LC3 plasmids to directly observe autophagic flux. Tetrandrine treatment significantly increased the colocalization of red and green fluorescence, appearing as yellow puncta, which indicates the impaired late stage of autophagic flux. This effect was comparable to that of CQ, another well-established inhibitor of autophagy at its terminal stages (Fig. 3e). To strengthen these findings, we compared tetrandrine’s effects to those of bafilomycin A1 (Baf) and investigated the effects of combining tetrandrine with either CQ or Baf. Remarkably, tetrandrine, CQ, and Baf all caused pronounced red-green colocalization, appearing as yellow puncta. Moreover, the combination of tetrandrine with either CQ or Baf did not further enhance the colocalization, confirming a robust inhibition of the late stage of autophagic flux across all treatment groups (Fig. S3). These results strongly indicate that tetrandrine acts similarly to CQ and Baf in blocking autophagic flux at the late stages, as evidenced by the substantial colocalization of red and green fluorescence.

Collectively, these insights affirm that tetrandrine’s enhancement of MHC-I expression in melanoma cells is likely mediated by its ability to disrupt the late stage of autophagic flux. This finding highlights tetrandrine’s potential as a novel strategy to modulate tumor immunogenicity and improve antitumor immune responses.

### Tetrandrine increases MHC-I levels by simultaneously inhibiting autophagy and proteasomal activity

**Fig. 4:**
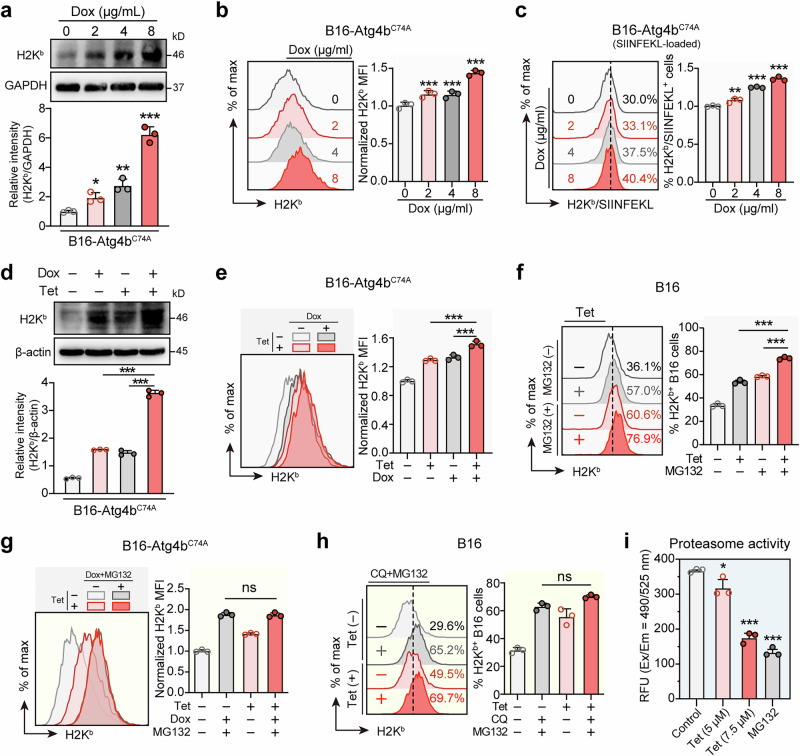
Tetrandrine increases MHC-I levels by simultaneously inhibiting autophagy and proteasomal activity. **a**, **b** Mutation of Atg4b blocks autophagy and increases the levels of both total and surface MHC-I (H2K^b^) in melanoma cells. B16-Atg4b^C74A^ cells, induced by Dox, express a mutant form of Atg4b that blocks autophagy. Cells were treated with varying concentrations of Dox (0, 2, 4, 8 μg/mL) for 24 h. Total MHC-I levels were assessed by Western blot, and surface MHC-I levels were measured using H2K^b^ fluorescent antibody staining and flow cytometry to determine mean fluorescence intensity. **c** Blocking autophagy with mutant Atg4b increases the level of MHC-I presentation. B16-Atg4b^C74A^ cells were incubated with SIINFEKL peptide and treated with varying concentrations of Dox (0, 2, 4, 8 μg/mL) for 24 h. Surface H2K^b^/SIINFEKL complex was assessed using fluorescent antibody staining and flow cytometry to measure mean fluorescence intensity. **d**, **e** Combined treatment with tetrandrine and Atg4b mutation, which blocks autophagy, leads to a further increase in both total and surface MHC-I in melanoma cells compared to individual treatments. B16-Atg4b^C74A^ cells were treated with vehicle, Dox (8 μg/mL), Tet (7.5 μM), or Dox+Tet for 24 h. Total MHC-I levels were analyzed by Western blot, and surface MHC-I levels were measured using H2K^b^ fluorescent antibody staining and flow cytometry. **f** Blocking proteasomal activity with MG132 leads to an increase in MHC-I levels, and combined treatment with tetrandrine and MG132 results in a further increase in MHC-I levels. B16 cells were treated with vehicle, MG132 (1 μM), Tet (7.5 μM), or MG132+Tet for 24 h. Surface MHC-I was assessed using FITC-H2K^b^ fluorescent antibody staining and flow cytometry. **g** Combined treatment with tetrandrine, MG132, and Dox does not lead to a further increase in MHC-I levels compared to MG132 and Dox combined. B16-Atg4b^C74A^ cells were treated with vehicle, MG132 (1 μM) combined with Dox (8 μg/mL), Tet (7.5 μM), or Tet+MG132+Dox for 24 h. Surface MHC-I was assessed using FITC-H2K^b^ fluorescent antibody staining and flow cytometry. **h** Combined treatment with tetrandrine, MG132, and CQ does not lead to a further increase in MHC-I levels compared to MG132 and CQ combined. B16 cells were treated with vehicle, MG132 (1 μM) combined with CQ (20 μM), Tet (7.5 μM), or Tet+MG132 + CQ for 24 h. Surface MHC-I was assessed using FITC-H2K^b^ fluorescent antibody staining and flow cytometry. **i** Tetrandrine treatment decreases proteasomal activity in melanoma cells. B16 cells were treated with vehicle, Tet (5, 7.5 μM), or MG132 (1 μM) for 24 h. Proteasomal activity was measured using a 20S proteasome activity assay kit. Tet tetrandrine, CQ chloroquine, Dox doxycycline. *P < 0.05, **P < 0.01, ***P < 0.001 indicate levels of statistical significance.

In exploring the effects of autophagy inhibition on MHC-I expression, we employed B16-Atg4b^C74A^ melanoma cells engineered to express a mutant form of Atg4b, which blocks autophagy when induced by doxycycline (Dox). Upon treatment with increasing concentrations of Dox, we observed a corresponding elevation in both total and surface MHC-I levels, confirming that autophagy inhibition via Atg4b mutation leads to enhanced MHC-I expression (Fig. 4a, b). Western blot analyses demonstrated this increase in total MHC-I (H2K^b^) levels (Fig. 4a), while flow cytometry validated a similar upregulation at the cell surface, showing elevated mean fluorescence intensity (Fig. 4b). Consistently, Atg4b mutation induced by doxycycline (Dox) also enhanced MHC-I (H2K^b^) mediated antigen presentation (Fig. 4c). Further investigation into combined treatments revealed that the simultaneous application of tetrandrine and the Atg4b mutation further enhanced MHC-I levels than the increase observed with either intervention alone (Fig. 4d, e). This suggests that inhibiting autophagy is not the only pathway through which tetrandrine enhances MHC-I levels.

Notably, the proteasomal pathway is a significant route for MHC-I degradation, and we sought to determine whether tetrandrine enhances MHC-I levels by inhibiting proteasomal activity. Our results showed that blocking proteasomal activity with MG132 alone led to an increase in MHC-I levels. When MG132 was combined with tetrandrine, we observed a further increase in MHC-I levels compared to MG132 treatment alone (Fig. 4f), indicating that tetrandrine contributes to MHC-I upregulation through an additional mechanism beyond proteasomal inhibition. To further investigate the interplay between autophagy and proteasomal inhibition, we performed experiments combining tetrandrine, MG132, and autophagy inhibition. Autophagy was inhibited through two complementary approaches: doxycycline (Dox)-activated Atg4b mutation, which specifically blocks autophagic flux upon Dox treatment, and chloroquine (CQ), a widely used inhibitor of late-stage autophagy. In both cases, the results showed that combined treatment with tetrandrine, MG132, and autophagy inhibition (induced by either Dox-activated Atg4b mutation or CQ) did not result in a further increase in MHC-I levels compared to the combination of MG132 and autophagy inhibition alone (Fig. 4g, h). This finding suggests that tetrandrine’s effect on MHC-I upregulation is mediated through its simultaneous inhibition of both autophagic flux and proteasomal activity, with no additional effect observed when both pathways are already maximally inhibited by MG132 and either Dox-activated Atg4b mutation or CQ treatment.

To further quantify tetrandrine’s contribution to MHC-I upregulation through the inhibition of these two degradation pathways, we examined its effects on surface H2K^b^ levels in B16 cells under combined treatments with either CQ or MG132. Flow cytometry analysis revealed that the increase in surface H2K^b^ levels in the Tet + CQ group compared to the CQ group could be attributed to tetrandrine’s inhibition of proteasomal activity. Conversely, the increase in the Tet + MG132 group compared to the MG132 group was attributed to tetrandrine’s inhibition of autophagy (Fig. S4). Interestingly, these results indicate that tetrandrine’s inhibition of autophagy makes a slightly greater contribution to the increase in MHC-I levels compared to its inhibition of proteasomal activity. Consistent with these observations, tetrandrine alone effectively decreased proteasomal activity in melanoma cells, as measured by a 20S proteasome activity assay (Fig. 4i). This finding further underscores its dual role in modulating degradation pathways to enhance MHC-I expression.

Collectively, these results demonstrate that tetrandrine enhances MHC-I levels through dual inhibition of autophagic and proteasomal degradation pathways. This multifaceted mechanism underscores tetrandrine’s potential to improve antigen presentation in melanoma cells, offering a promising strategy for boosting antitumor immunity.

### Tetrandrine inhibits the late stage of autophagic flux by impeding lysosomal acidification rather than inhibiting autophagosome-lysosome fusion

**Fig. 5:**
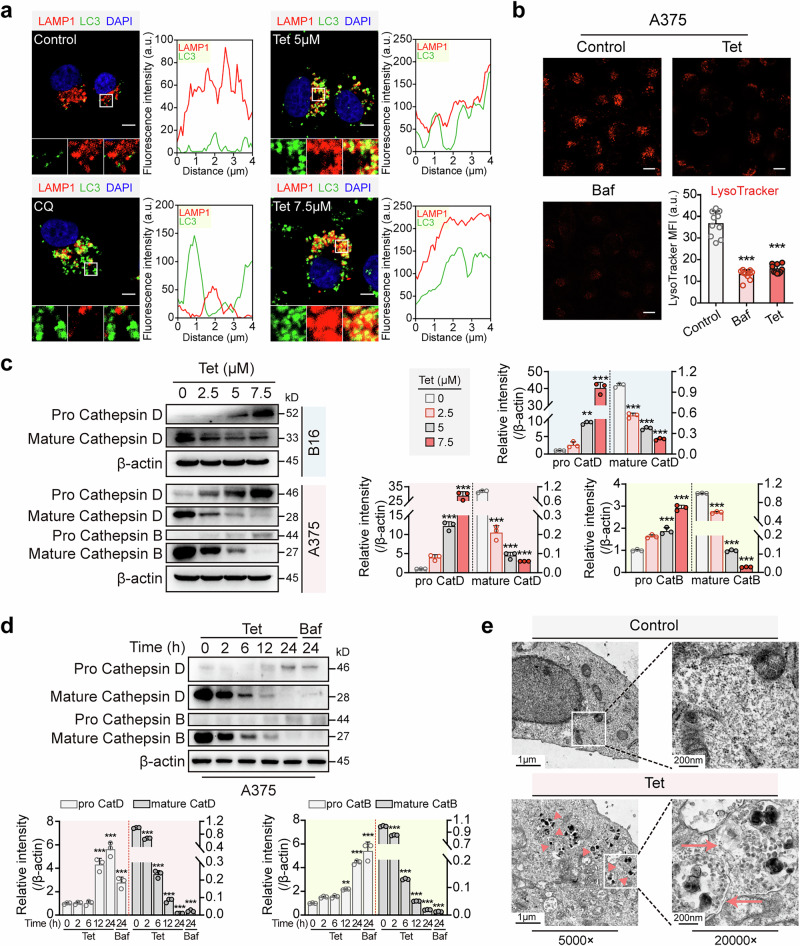
Tetrandrine inhibits the late stage of autophagic flux by impeding lysosomal acidification rather than inhibiting the fusion of lysosomes and autophagosomes. **a** A375 cells transfected with the hLAMP1-mCherry plasmid were exposed to tetrandrine (5 μM) or chloroquine (CQ, 20 μM) for 24 h. Following immunostaining for LC3-I/II (green), images were acquired using confocal microscopy, and colocalization between hLAMP1-mCherry (red) and LC3 (green) was analyzed using ImageJ software. Yellow fluorescence indicates the overlap of lysosomes and autophagosomes. The red and green lines in the figure correspond to the arbitrary units (a.u.) of red and green intensity within the rectangular region shown in the zoomed-in image. Scale bar: 5 μm. **b** Tetrandrine inhibits lysosomal acidification. A375 cells were treated with vehicle, tetrandrine (5 μM), or Baf (20 nM) for 24 h and subsequently stained with Lysotracker to evaluate lysosomal acidification. A decrease in red fluorescence intensity indicates suppressed lysosomal acidification, with both tetrandrine and Baf treatments showing a significant reduction in Lysotracker red fluorescence. Scale bar, 10 μm. **c**, **d** Tetrandrine inhibits the maturation of Cathepsins. A375 or B16 cells were treated with various concentrations of tetrandrine (0, 2.5, 5, 7.5 μM) for 24 h or with a fixed concentration of tetrandrine (5 μM) over different time points (0, 2, 6, 12, 24 h). Proteins were then extracted, and Western blot analysis was performed to assess Cathepsin maturation. Baf (20 nM) was used as a positive control. **e** Analysis of autophagosome-lysosome fusion by electron microscopy. A375 cells were treated with vehicle or tetrandrine (7.5 μM) for 24 h, and samples were collected for electron microscopy. As indicated by the red arrows, a higher number of autolysosomes were observed in the tetrandrine-treated group, suggesting that tetrandrine does not inhibit the fusion of autophagosomes and lysosomes. Tet tetrandrine, CQ chloroquine, Baf bafilomycin A1. ***P < 0.001 indicate levels of statistical significance.

We examined the effects of tetrandrine on late-stage autophagic flux in melanoma cells to determine whether its inhibitory action occurs through suppression of lysosomal acidification or autophagosome-lysosome fusion. First, we analyzed autophagosome-lysosome colocalization in A375 cells transfected with the hLAMP1-mCherry plasmid and treated with tetrandrine or chloroquine (CQ). Confocal microscopy revealed significant colocalization between lysosomes and autophagosomes, indicated by yellow fluorescence, suggesting that tetrandrine does not impair autophagosome-lysosome fusion (Fig. 5a). Similarly, we observed that bafilomycin A1 (Baf), a known lysosomal acidification inhibitor, also does not disrupt autophagosome-lysosome fusion (Fig. S5a), further confirming that neither Baf nor tetrandrine blocks this step of autophagy.

Next, we evaluated tetrandrine’s effect on lysosomal acidification using LysoTracker staining. A substantial reduction in red fluorescence was observed in tetrandrine-treated A375 cells, indicating a marked inhibition of lysosomal acidification, similar to the effect of bafilomycin A1 (Baf), a known inhibitor of this process (Fig. 5b). To further investigate the consequences of impaired lysosomal acidification, we assessed the maturation of lysosomal cathepsins, which require proper acidic conditions for maturation. Western blot analysis demonstrated that tetrandrine led to a concentration- and time-dependent decrease in cathepsin maturation in both A375 and B16 cells (Fig. 5c, d). Notably, qPCR analysis revealed that tetrandrine did not affect the transcriptional levels of cathepsins B and D, as shown in Fig. S5b. This indicates that the reduction in cathepsin maturation is likely due to tetrandrine’s disruption of lysosomal acidification rather than transcriptional regulation. Finally, we performed electron microscopy analysis to further examine tetrandrine’s effect on autophagosome-lysosome fusion. Tetrandrine treatment resulted in an increased number of autolysosomes, the structures formed by the fusion of autophagosomes with lysosomes, as indicated by red arrows. This observation further confirms that tetrandrine does not inhibit autophagosome-lysosome fusion (Fig. 5e).

In conclusion, these findings indicate that tetrandrine inhibits late-stage autophagic flux primarily by blocking lysosomal acidification, rather than disrupting autophagosome-lysosome fusion. This lysosomal dysfunction highlights tetrandrine’s potential as a therapeutic agent, particularly in enhancing melanoma immunogenicity through modulation of autophagic processes.

### Tetrandrine inhibits lysosomal acidification and cytosolic proteasomal activity by blocking the lysosomal calcium efflux channel TPC2

**Fig. 6:**
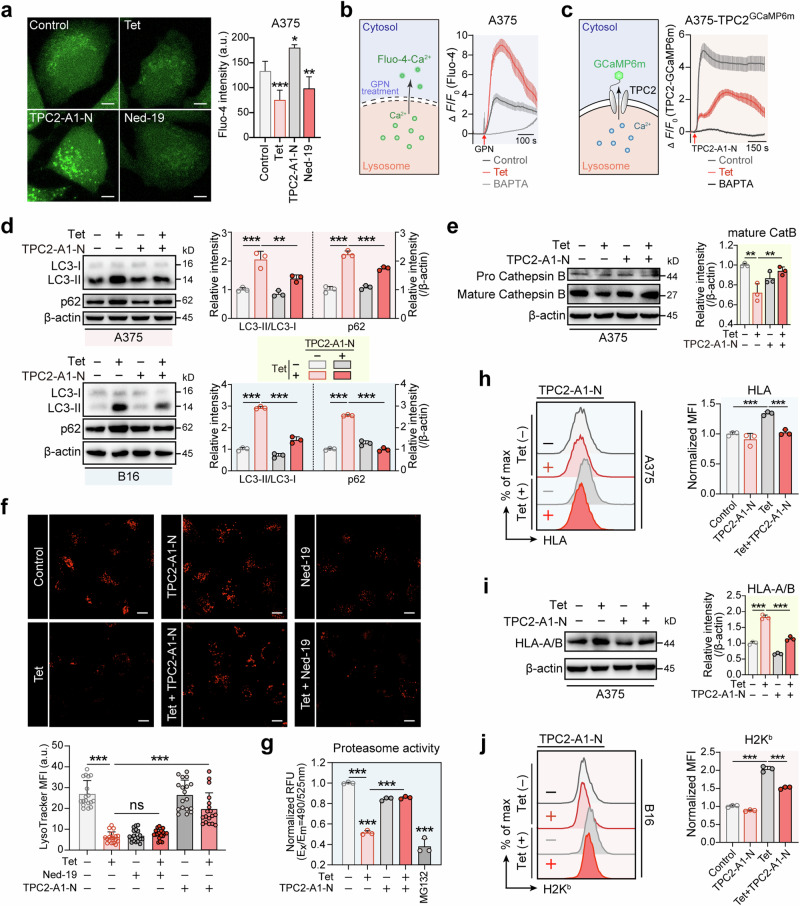
Tetrandrine inhibits lysosomal acidification and cytosolic proteasomal activity by blocking the lysosomal calcium efflux channel TPC2. **a** Tet reduces cytosolic calcium levels. A375 melanoma cells were treated with vehicle, Tet (7.5 μM), TPC2-A1-N (30 μM, TPC2 activator), or Ned-19 (50 μM, an antagonist of the endogenous TPC2 activator NAADP) for 6 h, followed by Fluo-4/AM staining (3 μM, 37°C, 30 min). Cytosolic calcium levels were measured using confocal microscopy based on green fluorescence intensity. Scale bar, 5 μm. **b** Tet increases lysosomal calcium levels. A375 melanoma cells were treated with vehicle, Tet (7.5 μM), or BAPTA-AM (5 μM, calcium chelator) for 6 h, followed by Fluo-4/AM staining (3 μM, 37°C, 30 min). GPN (400 μM) was then used to permeabilize lysosomes, and lysosomal calcium content was inferred from changes in cytosolic calcium measured by confocal microscopy. **c** Tet inhibits TPC2-mediated calcium release. A375 melanoma cells stably expressing TPC2-GCaMP6m, a calcium-sensitive reporter that emits green fluorescence upon TPC2 activation, were treated with vehicle, Tet (7.5 μM), or BAPTA-AM (5 μM) for 6 h. After Fluo-4/AM staining (3 μM, 30 min), TPC2-A1-N (30 μM) was added to stimulate TPC2, and calcium release was measured by confocal microscopy based on green fluorescence intensity. **d**, **e** TPC2 activation reverses Tet-induced autophagic flux blockade and Cathepsin B maturation inhibition. A375 melanoma cells were treated with vehicle, Tet (7.5 μM), TPC2-A1-N (30 μM), or Tet combined with TPC2-A1-N for 24 h. Western blot analysis showed that TPC2 activation reversed Tet-induced accumulation of LC3-II and P62, as well as the reduction in Cathepsin B maturation. Densitometric quantification of protein bands is presented in the bar graphs. **f** TPC2 activation restores lysosomal acidification inhibited by Tet. A375 melanoma cells were treated with vehicle, Tet (7.5 μM), TPC2-A1-N (30 μM), Tet + TPC2-A1-N, Ned-19 (50 μM), or Tet + Ned-19 for 6 h. Lysosomal acidification was assessed using LysoTracker staining, with red fluorescence intensity quantified by confocal microscopy in at least 15 cells per group. Scale bar, 10 μm. **g** TPC2 activation restores proteasomal activity inhibited by Tet. A375 melanoma cells were treated with vehicle, Tet (7.5 μM), TPC2-A1-N (30 μM), or Tet + TPC2-A1-N for 6 h. Proteasomal activity was measured using the Amplite® Fluorometric 20S Proteasome Assay Kit, with MG132 as a positive control. **h**, **i** TPC2 activation reverses Tet-induced upregulation of HLA expression in human melanoma cells. A375 melanoma cells were treated with vehicle, Tet (7.5 μM), TPC2-A1-N (30 μM), or Tet + TPC2-A1-N for 24 h. Surface HLA expression was measured by flow cytometry after PE-conjugated anti-HLA antibody staining, while total HLA-A/B protein levels were assessed by Western blot analysis. **j** TPC2 activation reverses Tet-induced upregulation of surface H2K^b^ in murine melanoma cells. B16 murine melanoma cells were treated with vehicle, Tet (7.5 μM), TPC2-A1-N (30 μM), or Tet + TPC2-A1-N for 24 h. Surface H2K^b^ expression was measured by flow cytometry following PE-conjugated anti-H2K^b^ antibody staining (20 min). BAPTA BAPTA-AM, Tet tetrandrine, TPC2 two-pore calcium channel 2. *P < 0.05, **P < 0.01, ***P < 0.001 indicate levels of statistical significance.

To further investigate the mechanism underlying tetrandrine-induced lysosomal dysfunction and proteasomal inhibition, we focused on calcium homeostasis, as tetrandrine has been previously identified as a blocker of TPC2 [18], a lysosomal calcium efflux channel [19]. Lysosomal calcium efflux is crucial for maintaining acidification by preserving the proton gradient necessary for lysosomal function, as shown in studies of other calcium channels [23]. Disruption of this efflux can cause calcium overload, leading to proton leakage and the breakdown of the acidic environment essential for lysosomal activity [24]. Additionally, cytosolic calcium is crucial for maintaining optimal proteasomal activity [20]. Based on these findings, we hypothesized that tetrandrine disrupts TPC2 function, resulting in calcium imbalance and subsequent impairment of lysosomal acidification and proteasomal activity. To test this hypothesis, we first examined the effects of tetrandrine on calcium homeostasis. We therefore examined whether tetrandrine disrupts TPC2 function, leading to calcium imbalance and subsequent impairment of these cellular processes. To confirm this hypothesis, we first confirmed the impact of tetrandrine on calcium homeostasis. Tetrandrine treatment significantly decreased cytosolic calcium levels while causing calcium accumulation within lysosomes in A375 melanoma cells, as demonstrated by Fluo-4/AM staining combined with GPN treatment, which permeabilizes lysosomes and releases stored calcium into the cytosol (Fig. 6a, b). Consistent with these findings, tetrandrine suppressed TPC2-mediated calcium release in TPC2-GCaMP6m-transfected cells, a calcium sensor that fluoresces upon TPC2 activation [25] (Fig. 6c). These data suggest that tetrandrine inhibits TPC2 activity, leading to lysosomal calcium retention and a reduction in cytosolic calcium levels. Next, we investigated whether this calcium imbalance was responsible for the observed lysosomal dysfunction and proteasomal inhibition. Western blot analysis revealed that tetrandrine treatment resulted in the accumulation of LC3-II and P62, along with a reduction in Cathepsin B maturation, consistent with impaired autophagic flux. Notably, co-treatment with the TPC2 activator TPC2-A1-N reversed these effects, restoring LC3-II and P62 levels while partially rescuing Cathepsin B maturation (Fig. 6d, e). Further investigation confirmed that tetrandrine impaired lysosomal acidification, as evidenced by a significant reduction in LysoTracker red fluorescence intensity. This effect was also reversed upon TPC2-A1-N co-treatment, indicating a restoration of lysosomal acidification to near-normal levels (Fig. 6f). Similarly, tetrandrine significantly reduced proteasomal activity, a suppression that was also reversed by TPC2 activation, as measured using the fluorometric 20S proteasome assay kit (Fig. 6g). Finally, we assessed whether the tetrandrine-induced disruption of calcium homeostasis influenced MHC-I expression. Flow cytometry and Western blot analysis demonstrated that tetrandrine treatment led to increased surface and total HLA expression in human A375 melanoma cells and elevated H2K^b^ expression in murine B16 melanoma cells. Importantly, these effects were reversed by TPC2 activation, further supporting the role of calcium imbalance and lysosomal dysfunction in tetrandrine-mediated MHC-I upregulation (Fig. 6h–j). These results collectively demonstrate that tetrandrine inhibits late-stage autophagic flux and proteasomal activity by blocking the lysosomal calcium efflux channel TPC2, thereby preventing MHC-I degradation and enhancing MHC-I-mediated antigen presentation.

### Tetrandrine inhibits melanoma growth through CD8^+^ T cell-mediated immune responses and enhances anti-PD-1 therapy efficacy

**Fig. 7:**
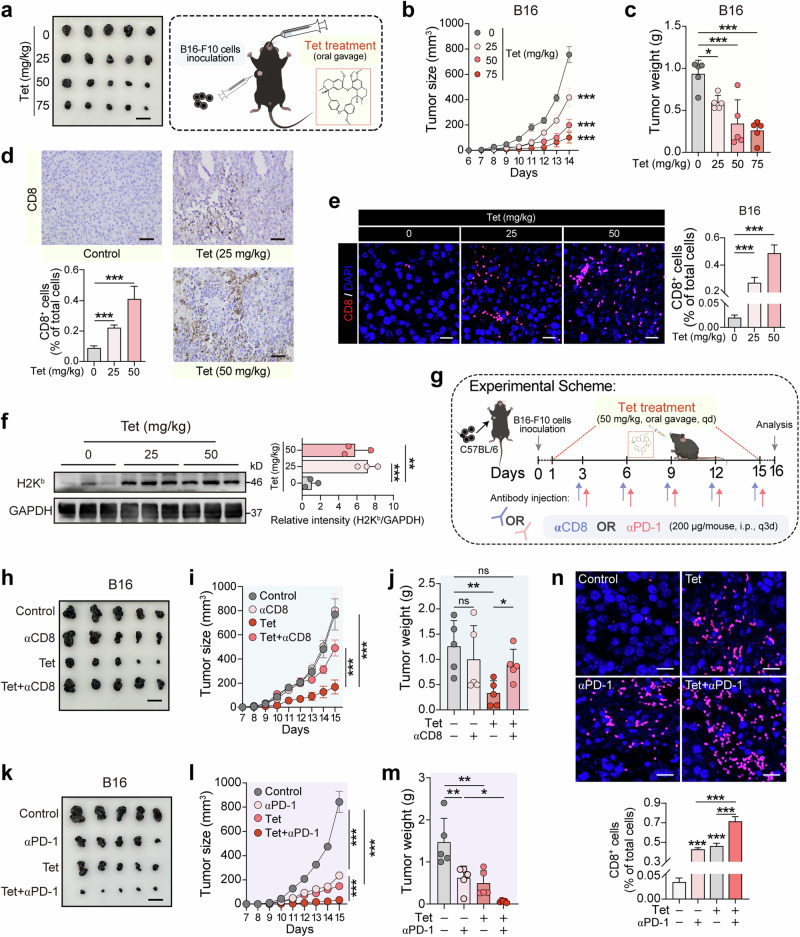
Tetrandrine inhibits melanoma growth through CD8^+^ T cell-mediated immune responses and enhances anti-PD-1 therapy efficacy. **a**–**c** Tumor growth curves **a** and tumor weight analysis **b** showing the inhibitory effect of tetrandrine on melanoma growth in vivo. C57BL/6 mice (*n* = 5 per group) were subcutaneously injected with 2 × 10^5^ B16 melanoma cells and were treated with varying concentrations of tetrandrine (0, 25, 50, 75 mg/kg). Tumor growth was monitored every day, at the end of the experiment, tumors were harvested and weighed. Scale bar, 2 cm. **d**, **e** Immunohistochemical and immunofluorescence analysis of CD8^+^ T cell infiltration in paraffin-embedded tumor tissues, indicating the percentage of CD8^+^ T cells. Scale bar, 50 μm **d** and 20 μm **e**. **f** Western blot analysis of H2K^b^ expression in tumor tissues from mice (*n* = 3 per group) treated with tetrandrine. **g** Schematic representation of CD8 depletion and anti-PD-1 combination therapy in animal experiments. **h**–**j** Tumor images **h**, tumor growth curves **i** and tumor weight analysis **j** showing the reversal of tetrandrine’s tumor inhibitory effect upon CD8 depletion. C57BL/6 mice were divided into groups: control, αCD8 (200 μg/mouse, i.p., every 3 days), Tet (50 mg/kg), and αCD8+Tet. Scale bar, 2 cm. **k**–**m** Tumor images **k**, tumor growth curves **l** and tumor weight analysis **m** showing the enhanced tumor inhibitory effect of tetrandrine in combination with anti-PD-1 therapy. C57BL/6 mice were divided into groups: control, αPD1 (200 μg/mouse, i.p., every 3 days), Tet (50 mg/kg), and αPD1+Tet. Scale bar, 2 cm. **n** CD8^+^ T cell infiltration in tumor tissues. Immunofluorescence analysis of tumor tissues from B16-F10 tumor-bearing C57BL/6 mice. The bar graph quantifies the percentage of CD8^+^ T cells among total cells, showing significantly increased infiltration in the Tet+αPD-1 group. Scale bar, 20 μm. Tet, tetrandrine. *P < 0.05, **P < 0.01, ***P < 0.001 indicate levels of statistical significance.

Building on our in vitro findings, we further explored tetrandrine’s antitumor effects in vivo. Using C57BL/6 mice implanted with B16 melanoma cells, we evaluated the impact of varying concentrations of tetrandrine on tumor progression and immune cell activation within the tumor microenvironment. Our in vivo experiments showed a clear inhibitory effect of tetrandrine on melanoma growth. Mice treated with tetrandrine at doses of 25, 50, and 75 mg/kg demonstrated progressively reduced tumor growth compared to controls, as evidenced by tumor growth curves and tumor weight measurements at the study’s conclusion (Fig. 7a–c). This dose-dependent inhibition highlights tetrandrine’s potential as a potent antitumor agent. To further understand the cellular mechanisms underlying these effects, we performed immunohistochemical and immunofluorescence analyses on tumor tissues. These studies revealed significant increases in CD8^+^ T cell infiltration in tumors from tetrandrine-treated mice, indicating an enhanced immune response (Fig. 7d, e). Western blot analysis of tumor tissues further supported these findings, showing increased expression of the MHC-I molecule H2K^b^, confirming tetrandrine’s role in promoting antigen presentation (Fig. 7f).

The role of CD8^+^ T cells in tetrandrine’s antitumor effect was first confirmed by depleting CD8^+^ T cells using anti-CD8 antibodies. To verify the efficiency and specificity of αCD8-mediated CD8^+^ T cell depletion, we performed flow cytometric analysis of splenocytes, as shown in Fig. S6. The gating strategy (Fig. S6a, b) confirmed that αCD8 treatment effectively reduced the percentage of CD3^+^ CD8^+^ T cells from 7.01% in the control group to 0.30% in αCD8-treated mice (Fig. S6c). Importantly, αCD8 treatment did not significantly affect other immune cell populations, such as CD45^+^ CD3^-^ cells or CD45^+^ CD3^+^ CD8^-^ cells, confirming the specificity of CD8^+^ T cell depletion without off-target effects (Fig. S6d). Following CD8^+^ T cell depletion, tetrandrine’s antitumor efficacy was significantly diminished, indicating that its inhibitory effect on melanoma growth is largely mediated by CD8^+^ T cell immune responses. Tumor growth curves and tumor weight analyses in these CD8-depleted groups demonstrated a reversal of tetrandrine’s inhibitory effects, further highlighting the critical role of CD8^+^ T cells in this process (Fig. 7g–j).

Subsequently, we explored the potential synergistic effect of combining tetrandrine with immune checkpoint blockade. Mice treated with a combination of tetrandrine and anti-PD-1 antibodies exhibited significantly enhanced tumor growth inhibition, suggesting a synergistic interaction between tetrandrine and immune checkpoint therapy (Fig. 7k–m). Immunofluorescence analysis of tumor tissues from B16-F10 tumor-bearing C57BL/6 mice further demonstrated significantly increased CD8^+^ T cell infiltration in the Tet+αPD-1 group, as quantified by the percentage of CD8^+^ T cells among total cells (Fig. 7n). These findings collectively demonstrate that tetrandrine inhibits melanoma growth through CD8^+^ T cell-mediated immune killing and enhances the efficacy of anti-PD-1 therapy. The data underscore the potential of using tetrandrine in combination with existing immunotherapies to improve treatment outcomes for melanoma patients.

## Discussion

**Fig. 8:**
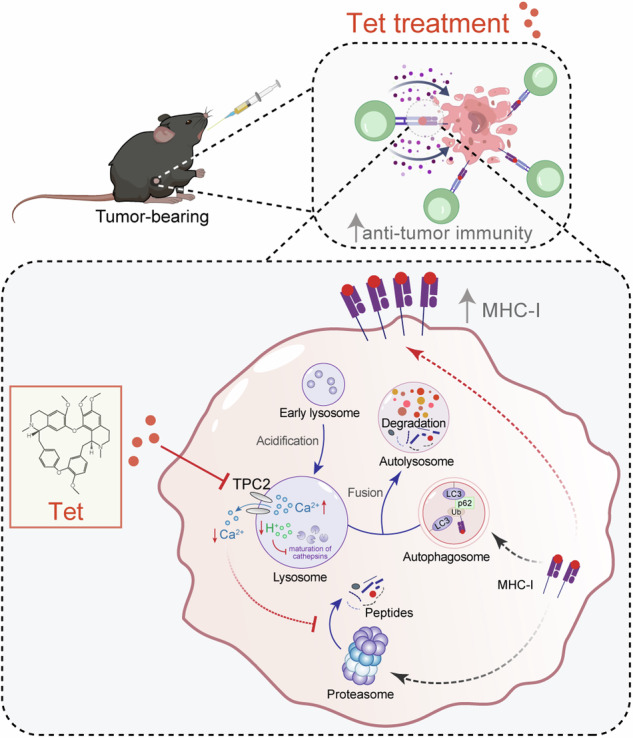
Mechanistic illustration of tetrandrine-mediated enhancement of melanoma cell recognition and killing by CD8^+^ T cells through the inhibition of autophagy and proteasomal activity. The diagram illustrates how MHC-I molecules in melanoma cells can be degraded through both autophagy and proteasomal pathways, leading to a reduction in surface MHC-I molecules and facilitating immune escape of the melanoma cells. Tetrandrine disrupts this degradation process by concurrently inhibiting late-stage autophagic flux, through lysosomal acidification disruption, and suppressing proteasomal activity. This dual inhibition prevents MHC-I degradation, thereby increasing MHC-I-mediated antigen presentation on the surface of melanoma cells. The elevated antigen presentation enhances CD8^+^ T cell recognition and cytotoxicity against melanoma cells. Further mechanistic exploration revealed that tetrandrine exerts its effects by blocking the lysosomal calcium efflux channel TPC2, leading to elevated lysosomal calcium levels and reduced cytosolic calcium concentrations. This calcium imbalance inhibits lysosomal acidification and suppresses cytoplasmic proteasomal activity, collectively contributing to reduced MHC-I degradation. The schematic emphasizes tetrandrine’s pivotal role in modulating both autophagic and proteasomal pathways, ultimately enhancing the immunogenicity of melanoma cells and increasing their susceptibility to CD8^+^ T cell-mediated cytotoxicity. Tet tetrandrine.

Our study provides a comprehensive understanding of how tetrandrine enhances MHC-I-mediated antigen presentation, presenting a novel strategy to augment antitumor immunity. As illustrated in Fig. 8, tetrandrine simultaneously inhibits autophagic flux and proteasomal activity, resulting in increased MHC-I stability and enhanced antigen presentation on melanoma cells. Specifically, tetrandrine disrupts lysosomal acidification and suppresses proteasomal activity by blocking the lysosomal calcium efflux channel TPC2. This inhibition leads to elevated lysosomal calcium levels and reduced cytosolic calcium concentrations, contributing to impaired autophagic degradation and proteasomal function. By preventing MHC-I degradation, tetrandrine promotes antigen presentation on the tumor cell surface, enhancing the recognition and cytotoxicity of CD8^+^ T cells against tumor cells. These findings highlight tetrandrine’s potential as a therapeutic agent targeting both autophagic and proteasomal pathways to overcome tumor immune escape and boost antitumor immunity.

MHC-I-mediated antigen presentation is crucial for antitumor immunity, as it enables the immune system to recognize and target tumor cells effectively [26, 27]. Our findings demonstrated that tetrandrine significantly upregulates MHC-I surface expression on melanoma cells in a dose-dependent manner, as confirmed by flow cytometry and Western blot analysis (Fig. 1c, d). Importantly, this upregulation is functional, as evidenced by the increased H2K^b^/SIINFEKL complex presentation (Fig. 1e, f), which improves CD8^+^ T cell recognition and cytotoxicity against tumor cells. These results position tetrandrine as a potent immunomodulatory agent that enhances tumor immunogenicity.

Autophagy is a well-established pathway involved in the degradation and recycling of cellular components, including MHC-I molecules [13, 28]. Research indicates that the autophagic process involves the formation of autophagosomes, which fuse with lysosomes to degrade their contents, thereby affecting the recycling and degradation of MHC-I molecules [29, 30]. One of the key mechanisms by which tetrandrine enhances MHC-I expression is through the inhibition of autophagic flux. Our experiments using A375-GFP-LC3 cells revealed a significant accumulation of autophagosomes upon tetrandrine treatment (Fig. 3a). Autophagy involves sequestering cytoplasmic content into double-membraned autophagosomes, which then fuse with lysosomes to form autolysosomes where the content is degraded [31]. The inhibition of autophagy by tetrandrine was further evidenced by increased levels of P62 and LC3-II, markers of autophagosome accumulation (Fig. 3b, c). Notably, tetrandrine’s inhibition of autophagy was associated with the suppression of lysosomal acidification rather than disrupting autophagosome-lysosome fusion (Fig. 5). This specific modulation of the autophagic pathway highlights tetrandrine’s potential in altering cellular degradation processes to favor MHC-I stability and presentation.

In addition to autophagy, proteasomal degradation is another critical pathway regulating MHC-I turnover and stability [12]. The proteasome degrades misfolded or damaged proteins, including MHC-I molecules. Inhibition of proteasomal activity can lead to the accumulation and enhanced stability of MHC-I molecules, improving their surface expression [12]. In addition to inhibiting autophagy, tetrandrine also impedes proteasomal activity, increasing the stability and abundance of MHC-I molecules (Fig. 4). Our data showed that tetrandrine treatment significantly reduced proteasomal activity in melanoma cells (Fig. 4i). Further analysis showed that melanoma cells treated with tetrandrine, in the context of combined inhibition of autophagy and proteasomal degradation, did not exhibit additional increases in MHC-I levels. This indicates that tetrandrine elevates MHC-I levels through the simultaneous inhibition of both autophagy and proteasomal activity (Fig. 4g, h). This dual inhibition mechanism is critical for maintaining higher levels of MHC-I molecules on the cell surface, thereby improving antigen presentation and immune recognition.

Our findings also provide new insights into the mechanisms underlying tetrandrine-induced lysosomal dysfunction and proteasomal inhibition, highlighting the critical role of calcium homeostasis in these processes. By blocking TPC2, a lysosomal calcium efflux channel, tetrandrine disrupts the normal release of calcium from lysosomes, leading to calcium accumulation within lysosomes and a concurrent reduction in cytosolic calcium levels (Fig. 6a–c). This calcium imbalance is central to the observed effects. Normal lysosomal calcium efflux is required to maintain lysosomal acidification [23], while lysosomal calcium overload can result in proton leakage [24], thereby disrupting the acidic environment necessary for proper lysosomal function. Furthermore, cytosolic calcium is essential for optimal proteasomal activity [20]. The tetrandrine-induced disruption of calcium homeostasis impairs lysosomal acidification and proteasomal function, leading to the stabilization of MHC-I molecules on the cell surface by preventing their degradation. Importantly, co-treatment with the TPC2 activator TPC2-A1-N restored lysosomal acidification, proteasomal activity, and MHC-I degradation, confirming the role of TPC2 in tetrandrine’s mode of action (Fig. 6d–j). These findings underscore the importance of calcium signaling in regulating key cellular degradation pathways and identify TPC2 as a promising therapeutic target for enhancing MHC-I-mediated antigen presentation and mitigating tumor immune escape.

The relationship between MHC-I and antitumor immunity is well-documented, with MHC-I playing a critical role in presenting tumor antigens to CD8^+^ T cells, thus initiating and sustaining effective immune responses against cancer cells [32, 33]. The functional significance of tetrandrine-induced MHC-I upregulation was further validated by in vitro and in vivo experiments demonstrating enhanced CD8^+^ T cell recognition and killing of melanoma cells (Figs. 2, 7). Co-culture assays with CD8^+^ T cells from OT-I mice showed a significant increase in T cell-mediated cytotoxicity against tetrandrine-treated melanoma cells (Fig. 2b), accompanied by increased IFN-γ secretion, reflecting heightened T cell activation (Fig. 2c). Flow cytometric analysis further confirmed a higher proportion of apoptotic melanoma cells in the presence of tetrandrine, reinforcing its role in boosting T cell-mediated immune responses (Fig. 2d). Our in vivo studies further underscored tetrandrine’s potential as an effective antitumor agent (Fig. 7). Tetrandrine treatment significantly inhibited melanoma growth in C57BL/6 mice, showing a dose-dependent reduction in tumor size and weight (Fig. 7a–c). Immunohistochemical and immunofluorescence analyses revealed increased infiltration of CD8^+^ T cells in tetrandrine-treated tumors, indicating an enhanced immune response within the tumor microenvironment (Fig. 7d, e). Importantly, CD8^+^ T cell depletion significantly reversed the antitumor effects of tetrandrine, confirming its reliance on CD8^+^ T cell-mediated immune killing (Fig. 7h–j). Moreover, tetrandrine’s ability to increase CD8^+^ T cell infiltration suggests a potential synergistic interaction with immune checkpoint blockade. The combination of tetrandrine with anti-PD-1 therapy led to significantly enhanced tumor growth inhibition, underscoring tetrandrine’s potential to improve the efficacy of existing immunotherapies (Fig. 7k–m). The combination of tetrandrine with anti-PD-1 significantly increased CD8^+^ T cell infiltration, as shown by immunofluorescence analysis (Fig. 7n), reinforcing its potential to boost antitumor immunity.

Tetrandrine, a bis-benzylisoquinoline alkaloid derived from the plant *Stephania tetrandra*, possesses a broad spectrum of pharmacological activities, including anti-inflammatory, anti-fibrotic, and anti-cancer effects [34]. However, its interaction with multiple cellular targets raises the potential for off-target effects. For example, tetrandrine modulates various signaling pathways, such as those involved in inflammation and fibrosis, contributing to its therapeutic effects but also posing risks of unintended interactions [35]. Given its diverse biological activities, tetrandrine may influence other cellular processes beyond its intended targets. Notably, its role as a calcium channel blocker could affect calcium-dependent pathways, potentially leading to unforeseen physiological consequences. While tetrandrine demonstrates significant promise as a therapeutic agent, particularly in oncology [36], a more comprehensive understanding of its pharmacological profile is necessary. Further studies are needed to investigate its off-target effects and ensure both its safety and efficacy in clinical applications.

Taken together, our findings demonstrate that tetrandrine enhances MHC-I-mediated antigen presentation by simultaneously inhibiting autophagy and proteasomal activity. This dual mechanism improves CD8^+^ T cell recognition and cytotoxicity, leading to significant antitumor effects both in vitro and in vivo. Moreover, tetrandrine synergizes with anti-PD-1 therapy, highlighting its potential as an adjunct in cancer immunotherapy. Future studies should investigate its broader applicability across different cancer types and explore its potential in combination with other immunotherapeutic strategies to further enhance treatment outcomes.

## Supplementary information

Supplementary materials

## Data Availability

The data that support the findings of this study are available from the corresponding author upon reasonable request.
