# Supplementary material for: Tetrandrine augments melanoma cell immunogenicity via dual inhibition of autophagic flux and proteasomal activity enhancing MHC-I presentation
Source: Acta Pharmacol Sin. 2025 Feb 27;46(7):2056–72. doi: 10.1038/s41401-025-01507-9 (PMC12205077; doi:10.1038/s41401-025-01507-9)
Supplement: Supplementary file 1 — Supplementary materials [file 41401_2025_1507_MOESM1_ESM.docx]

Supplementary Materials for

**Tetrandrine augments melanoma cell immunogenicity via dual inhibition of autophagic flux and proteasomal activity enhancing MHC-I presentation**

Lina He, Yujiao Liu, Junbo Jiang, Dingye Wang, Yuling Li, Shiji Zeng, Zi Guo, Peiyan Yao, Zichang Lin, Sixian Lv, Xiaoyi Liu, Wei Guo, Fang Liu, Tingxiu Zhao, Jianyong Xiao, Yafei Shi, Kun Wang

Corresponding author: wangkun@gzucm.edu.cn (K.W.); shiyafei@gzucm.edu.cn (Y.S.); jianyongxiao@gzucm.edu.cn (J.X.); zhaotingxiu@gzucm.edu.cn (T.Z.).

**The PDF file includes:**

Materials and Methods

Figs. S1 to S6

**Materials and Methods**

***CCK8 Assay for Cell Viability***

B16 melanoma cells were seeded in 96-well plates at 5,000 cells per well and allowed to adhere overnight. The next day, cells were treated with tetrandrine at concentrations of 0, 2.5, 5, and 7.5 μM. After 24 hours of treatment, cell viability was assessed using the CCK8 assay (Dojindo Laboratories). Briefly, 10 μL of CCK8 solution was added to each well and incubated for 2 hours at 37°C. Absorbance at 450 nm was measured using a microplate reader. Cell survival was calculated as a percentage of the control (untreated) cells.

***Quantitative PCR (qPCR) Analysisy***

Cells were seeded in 6-well plates and allowed to adhere overnight. The next day, cells were treated with tetrandrine at indicated concentrations for 24 hours. Total RNA was extracted using TRIzol reagent (Thermo Fisher Scientific, Cat. No. 15596018CN) and reverse-transcribed into cDNA using the Evo M-MLV RT Kit (Accurate Biology, Cat. No. AG11706). Quantitative PCR (qPCR) was performed using SYBR Green Master Mix (Accurate Biology, Cat. No. AG11740) on The StepOne Real-Time PCR System (Applied Biosystems™). Primers specific for human HLA-A, HLA-B, Cathepsin B, Cathepsin D, and mouse H2-K1, H2-D1, Cathepsin D were used. Relative mRNA levels were normalized to GAPDH and calculated using the 2^-ΔΔCt^ method.

**Supplementary Figures**

**
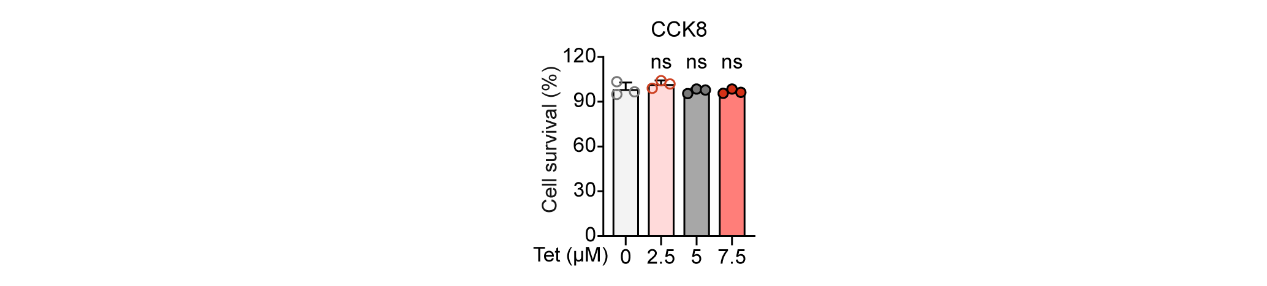
**

**Fig. S1. Tetrandrine does not induce cytotoxicity in B16 melanoma cells.** B16 melanoma cells were treated with varying concentrations of tetrandrine (0, 2.5, 5, and 7.5 μM) for 24 hours. Cell viability was assessed using the CCK8 assay. The results indicate no significant cytotoxic effects of tetrandrine on B16 cells at the tested concentrations. ns, no statistical significance.


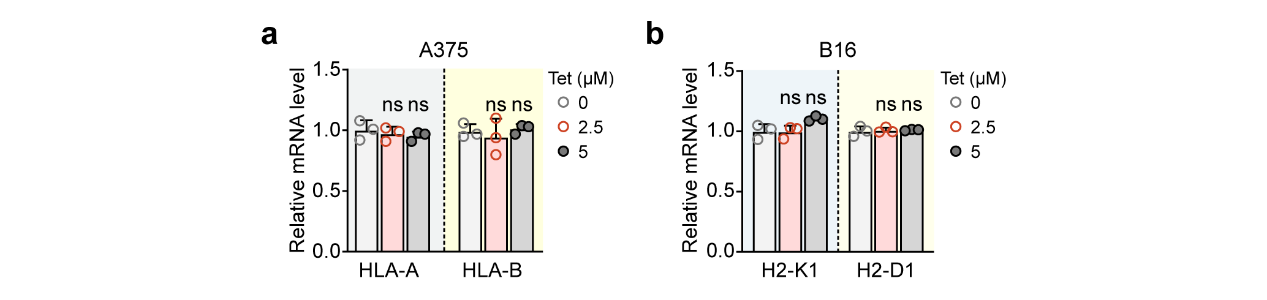


**Fig. S2. Tetrandrine does not affect the expression levels of genes responsible for MHC-I.** (**a**) Relative mRNA levels of HLA-A and HLA-B in A375 cells treated with tetrandrine at concentrations of 0, 2.5, and 5 μM. (**b**) Relative mRNA levels of H2-K1 and H2-D1 in B16 cells treated with tetrandrine at concentrations of 0, 2.5, and 5 μM. qPCR analysis indicates no significant changes in the expression of these MHC-I genes across different tetrandrine treatments. ns, no statistical significance.


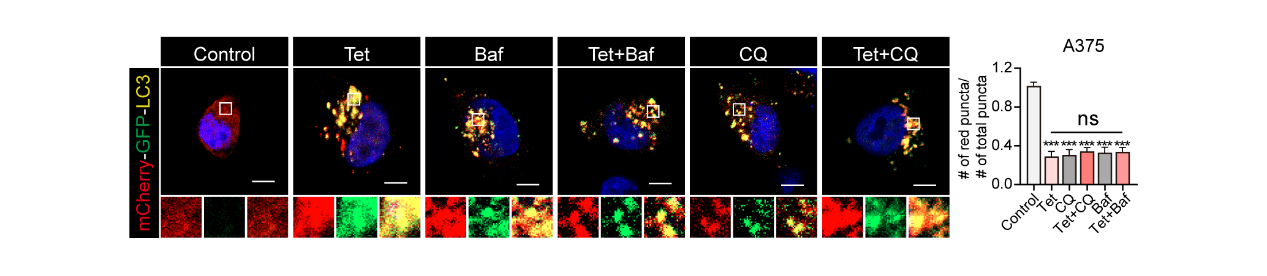


**Fig. S3. Tetrandrine treatment enhances the colocalization of red and green fluorescence in mCherry-GFP-LC3-transfected melanoma cells, indicative of late-stage autophagic flux blockade.** A375 cells transfected with mCherry-GFP-LC3 plasmids were treated with vehicle, Tet (5 μM), Baf (20 nM), Tet + Baf, CQ (20 μM), or Tet+CQ for 24 hours. Confocal microscopy was used to assess the presence of yellow puncta, indicative of impaired late autophagic flux due to colocalization of red and green fluorescence. Compared to the control group, all treatment groups exhibited a significant increase in red-green colocalization, appearing as yellow puncta. Scale bar, 5 μm. Tet, tetrandrine; CQ, chloroquine; Baf, bafilomycin A1. ***p < 0.001 indicates levels of statistical significance. ns, no statistical significance.


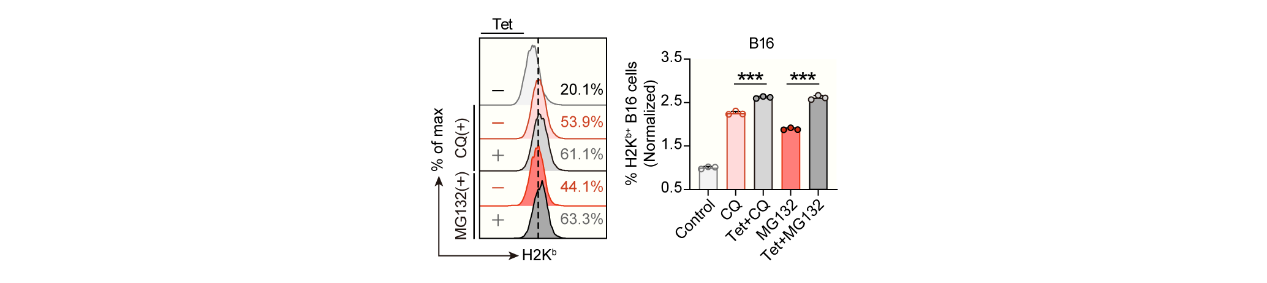


**Fig. S4. Tetrandrine enhances surface H2K^b^ levels by inhibiting both proteasomal and autophagic degradation pathways.** B16 cells were treated with vehicle, CQ (20 μM), Tet (7.5 μM) +CQ, MG132 (1 μM), or Tet+MG132 for 24 hours. Surface H2K^b^ levels were quantified by flow cytometry following PE-conjugated anti-H2K^b^ antibody staining (20 min). The increase in surface H2K^b^ levels in the Tet+CQ group compared to the CQ group is attributed to tetrandrine’s inhibition of proteasomal activity, while the increase in the Tet+MG132 group compared to the MG132 group is attributed to tetrandrine’s inhibition of autophagy. ***p < 0.001 indicates levels of statistical significance.


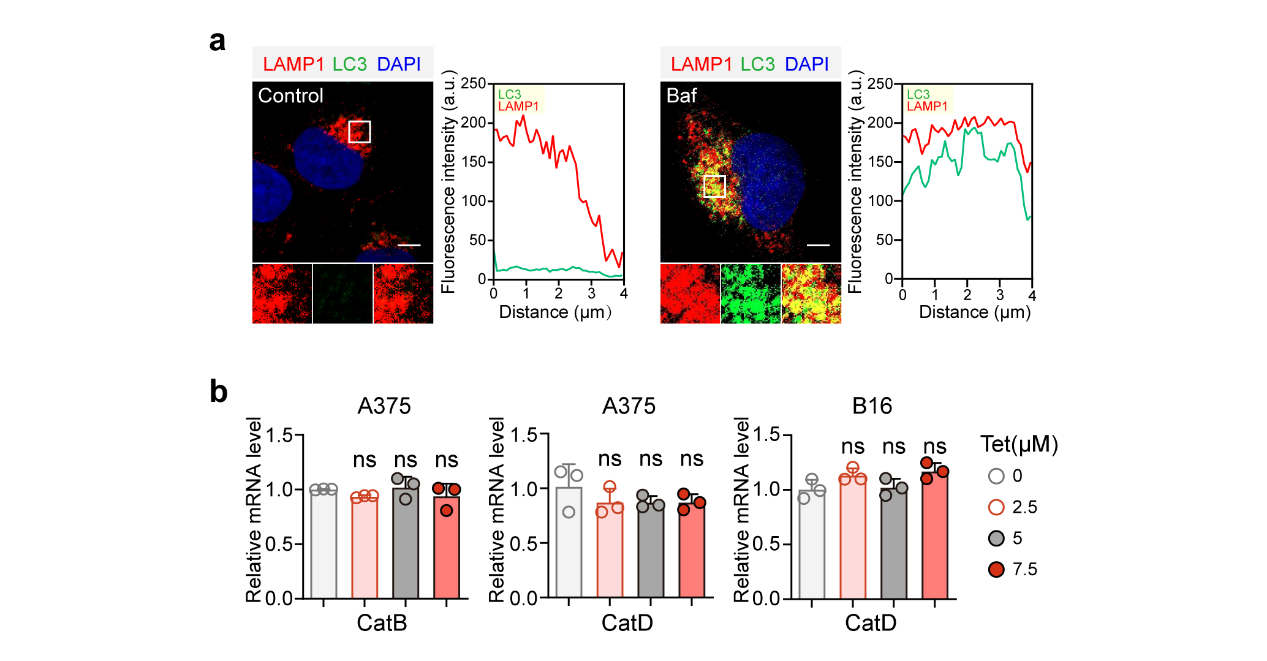


**Fig. S5. Bafilomycin A1 does not inhibit autophagosome-lysosome fusion, similar to tetrandrine, and tetrandrine does not affect the transcriptional levels of Cathepsins in melanoma cells.** (**a**) Supplement to Fig. 5A, showing the effect of Baf on autophagosome-lysosome fusion. A375 cells transfected with the hLAMP1-mCherry plasmid were treated with vehicle or Baf (20 nM) for 24 hours. Following immunostaining for LC3-I/II (green), images were captured using confocal microscopy, and colocalization between hLAMP1-mCherry (red) and LC3 (green) was analyzed using ImageJ software. Yellow fluorescence indicates the overlap of lysosomes and autophagosomes. The red and green lines in the figure represent the arbitrary units (a.u.) of red and green intensity within the rectangular region highlighted in the zoomed-in image. Scale bar: 5 μm. (**b**) A375 and B16 cells were treated with various concentrations of tetrandrine (0, 2.5, 5, 7.5 μM) for 24 hours. qPCR analysis showed no significant changes in the transcriptional levels of cathepsins across different tetrandrine treatments. Tet, tetrandrine; Baf, bafilomycin A1; CatB, cathepsin B; CatD, cathepsin D. ns, no statistical significance.


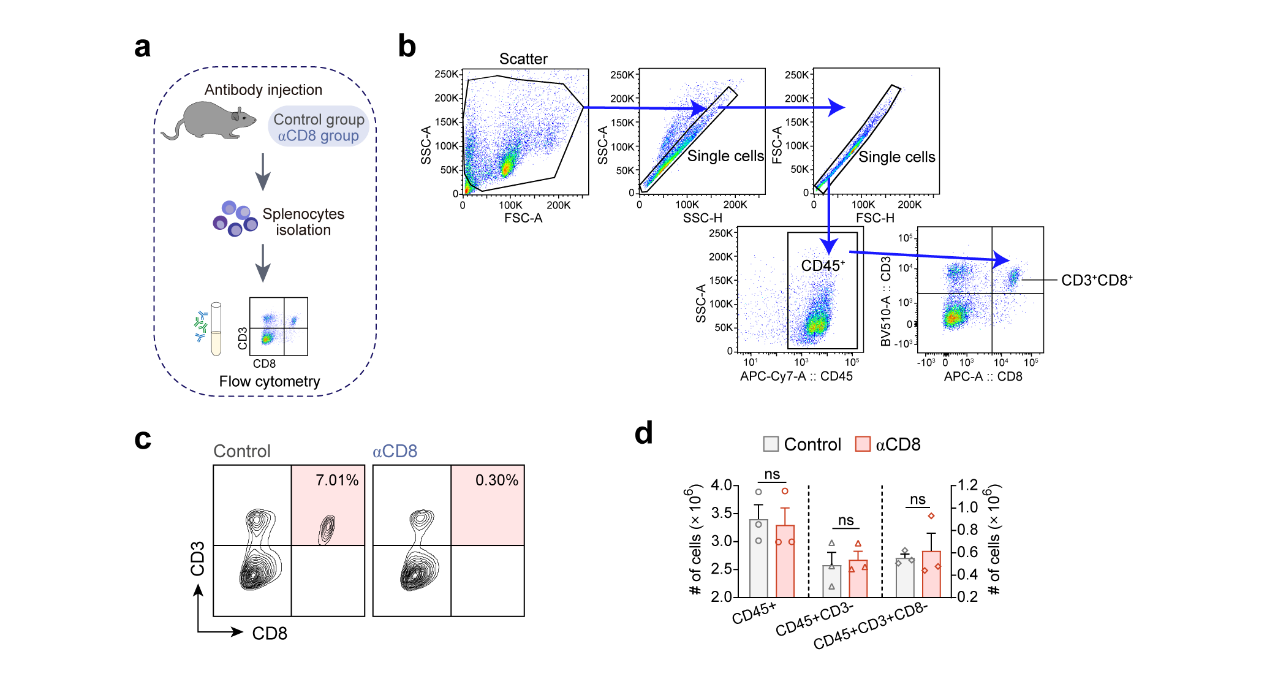


**Fig. S6. Evaluation of αCD8-mediated depletion of CD8^+^ T cells in mice.** (**a**) Schematic representation of the experimental procedure for assessing the efficacy of αCD8-mediated CD8^+^ T cell depletion in mice by analyzing splenocytes via flow cytometry. (**b**) Gating strategy for identifying CD8^+^ T cells. Splenocytes were gated sequentially on scatter properties, single cells, CD45^+^ cells, and finally CD3^+^ CD8^+^ T cells. (**c**) Density plots showing the depletion of CD8^+^ T cells in αCD8-treated mice compared to control mice. The percentage of CD3^+^ CD8^+^ T cells in splenocytes was reduced from 7.01% in the control group to 0.30% in the αCD8-treated group. (**d**) Quantification of splenic immune cell populations showing that αCD8 treatment did not significantly affect the total number of CD45^+^ cells, CD45^+^CD3^-^ cells, or CD45^+^ CD3^+^ CD8^-^ cells. ns, no statistical significance.
